# Ilizarov method and its combined methods in the treatment of long bone defects of the lower extremity: systematic review and meta-analysis

**DOI:** 10.1186/s12891-023-07001-9

**Published:** 2023-11-16

**Authors:** Lijun Xie, Ye Huang, Libi Zhang, Shuting Si, Yunxian Yu

**Affiliations:** 1 Department of Orthopedic Surgery, the Second Affiliated Hospital, Zhejiang University School of Medicine, Hangzhou City, Zhejiang Province P. R. China; 2 Orthopedics Research Institute of Zhejiang University, Hangzhou City, Zhejiang Province P. R. China; 3 Key Laboratory of Motor System Disease Research and Precision Therapy of Zhejiang Province, Hangzhou City, Zhejiang Province P. R. China; 4 Clinical Research Center of Motor System Disease of Zhejiang Province, Hangzhou City, Zhejiang Province P. R. China; 5 Department of Public Health, and Department of Anesthesiology, Second Affiliated Hospital of Zhejiang University School of Medicine, Hangzhou City, Zhejiang Province P. R. China; 6 Department of Epidemiology & Health Statistics, School of Public Health, School of Medicine, Zhejiang University, Hangzhou City, Zhejiang Province P. R. China

**Keywords:** Bone disease, Distraction osteogenesis, Femur, Internal fixators, Tibia

## Abstract

**Background:**

Ilizarov method has become one of primary methods for treating bone defects. Currently, there is growing trend in the application of modified Ilizarov methods (e.g., applying unilateral external fixators or with flap tissue) and its combined methods (e.g., Ilizarov method with antibiotic spacer or internal fixation) to manage bone defects. However, there is a lack of studies with systematical evaluation of the clinical effects of these evolving methods. This study aimed to conduct a systematic review and meta-analysis for overall evaluating the clinical effects on long bone defects of lower extremity in Ilizarov methods and its combined methods.

**Methods:**

Studies were identified in three electronic databases (Pubmed, Embase and Cochrane Library) from the earliest indexing year through November 01, 2022, and relevant data were extracted subsequently. The total number of participants, number of participants with bone unions, bone result or functional result, and related complications including pin infection, pin loosening, pain, refracture, limb discrepancy, malalignment, joint stiffness, recurrent infection, and amputation were extracted in this study. Then, union rate (defined as the proportion of patients who achieved bone unions) and specific complication incidence rate (defined as the proportion of patients who experienced specific complication) were pooled estimated respectively. Relative risk (RR) was used for comparing the clinical effects among various Ilizarov technique.

**Results:**

Sixty-eight case series studies, 29 comparative studies, and 3 randomized clinical trials were finally included. The union rate of Ilizarov methods was 99.29% (95% CI: 98.67% ~ 99.86%) in tibial defects and 98.81% (95% CI: 98.81% ~ 100.00%) in femoral defects. The union rate of Ilizarov method with antibiotic spacer and intramedullary nail in tibial defects was 99.58% (95% CI: 98.05% ~ 100.00%) and 95.02% (95% CI: 87.28% ~ 100.00%), respectively. Compared to the Ilizarov methods, the union rate of the Ilizarov method with antibiotic spacer in tibial defects increased slightly (RR = 1.02, 95% CI: 1.01 ~ 1.04). Meanwhile, compared to Ilizarov methods, we found lower excellent rate in bone result in Ilizarov method with antibiotic spacer, with the moderate to high heterogeneity. Compared to the Ilizarov method, lower rate of pin infection, higher rate of recurrent infection and amputation were observed in Ilizarov method with intramedullary nail, however, the findings about the comparison of pin infection and recurrent infection between the two groups were presented with high degree of statistical heterogeneity.

**Conclusion:**

Our study confirmed the reliable treatment of Ilizarov methods and its combined technique on long bone defects, and founded there were significant differences on some complications rate between Ilizarov methods and its combined technique. However, the findings need to be confirmed by further studies.

**Supplementary Information:**

The online version contains supplementary material available at 10.1186/s12891-023-07001-9.

## Introduction

Nonunion with bone loss is common in clinical practices, frequently as consequence of trauma, osteomyelitis or tumour resection, and often poses extremely challenging problems for clinical surgeons when infection or soft tissue defects occurred in bone defect patients. Several methods have been advocated in the last decades, such as allogenic grafting, cancellous autograft, the Papineau technique, vascularized bone grafts, Masquelet technique and the Ilizarov method [1–6].

The Ilizarov technique (known as distraction osteogenesis), developed by Gavril Abramovich Ilizarov in 1950, has revolutionized the management of long bone defects [6]. This method primarily involves the use of an external fixator device, which comprises metal rings and wires, to stabilize and gradually separate the bone ends. The gradual distraction of the bone creates a controlled environment that stimulates new bone formation, allowing for precise and predictable bone lengthening. Up to now, numerous studies have reported the desired union rate when managing bone defects, such as the fractures or infected union [7–9], through the use of the Ilizarov bone transport technique. This method is conducted by gradually translocating segment of bone from healthy side to the region of bone loss [10]. For bone defects, the Ilizarov technique can theoretically be applied for the reconstruction of any length, and offers less invasive fashion approach and greater versatility when compared to other technique, especially for large bone reconstruction [6, 11, 12]. Gradually, Ilizarov method have become one of main methods for treating bone defects. However, there were still many complications with high incidence rate associated with Ilizarov methods, including pin infections, refractures, especially in cases with prolonged external fixation [7, 12]. Besides, the method involves the application of external fixator device comprising rings and wires, which can pose technically challenging and cause discomfort and inconvenience for patients [12, 13].

Currently, the original Ilizarov method has been modified in various ways. For example, unilateral external fixators were applied for replacing the heavy and cumbersome original ring frame [14, 15]. Besides, given that remaining wound opening during the operation of Ilizarov technique may be associated with infection, the free tissue with Ilizarov method were posed in some clinical practice [12, 16–18]. For shortening the long external fixation time (EFT), a new combined technique of segmental transport over an intramedullary nail was developed by Raschke et al. in 1982 [19] and used for reconstructing bone defect [20, 21]. The intramedullary nail is inserted into the medullary canal of the bone, acts as an internal reinforcement, which contributes to the overall strength and stability of the lengthened bone for shortening the long EFT [22]. In addition, with the purpose of reducing the infection, some studies reported the combined use of antibiotic spacer and bone transport [23, 24]. This innovative approach integrates the principles of distraction osteogenesis with the antibiotic-loaded spacers, which allows for high concentrations of antibiotics to be delivered directly to the infected site and may effectively tackle the underlying infection.

Although the application of modified Ilizarov methods and its combined methods for bone defects increased in recent years, the previous systematic reviews and meta-analyses were primarily focused on all studies involving the traditional Ilizarov technique or often overlooked to address its modifications [7, 25, 26]. Notably, the modified and combined technique of Ilizarov method have not been adequately covered in prior reviews. To date, only one systematic review has assessed the effects of Ilizarov method combined with antibiotic spacers for the management of infectious bone defects, and it primary reported the rates of bone union and reinfection [27]. Thus, there is need to comprehensively evaluate the effects of modified Ilizarov method and its combined technique to enhance more understanding of this field. To address this gap, we conducted a systematic review and meta-analysis to obtain the overall evaluation and comparison of clinical effects among various Ilizarov methods. Our primary focus was on the management of long bone defects in the lower extremity due to limitations in the number of available studies that we identified.

## Materials and methods

### Data sources and search strategies

We performed comprehensive searches in PubMed (Medline), Embase, and the Cochrane Library from the earliest indexing year of each database through November 01 2022. The following search terms and Boolean operators were used to identified relevant articles: bone AND (loss OR defect OR nonunion OR malunion) AND (Ilizarov OR “bone transport*” OR “distraction osteogenesis”). In addition, we further searched potentially relevant articles in the citation lists of eligible studies and review articles published. This study was conducted in accordance with the Preferred Reporting Items for Systematic Reviews and Meta-analysis (PRISMA) guidelines.

### Inclusion and exclusion criteria

We searched for all randomized controlled trials, prospective studies, retrospective studies and case series. The articles conducted among patients aged 16–70 and written in English were retained. The original articles were included where the main focus of the article was the management of osseous defects (caused by infection or non-infection) of the long bones of the lower extremities (tibia or femur), and the methods of management were the conventional Ilizarov method, its modified methods (e.g., Ilizarov methods by unilateral frame or with flap) or its combined methods (e.g., Ilizarov method with antibiotic spacer or internal fixation). The study reported two following outcomes (common index for assessing clinical effect) at least were seem eligible: union rate; bone results or functional results estimated by Association for the Study of the Method of Ilizarov (ASAMI) criteria; complications associated with the treatment; EFT and external fixation index (EFI). In addition, when duplicate publications were identified, the report of the largest number of cases were retained. The articles were included when references were only available in abstract form while data were extracted in sufficient detail. In cases where one group within a study met the inclusion criteria, we treated that group as an independent study and referred to it as a "sub-study" within the context of this research.

Studies were excluded if the sample size was less than 10; if only contained animal models or experiments; if bone defects incidence of study population was not 100% or the specific data of patients with bone defects was unable to extract from the original article; if bone defect was caused by malignancies or lengthening limb surgery; if evaluated femoral and tibial bone defects together and it was impossible to extract data separately; if type of article was letter or review; if full article was unavailable.

**Fig. 1:**
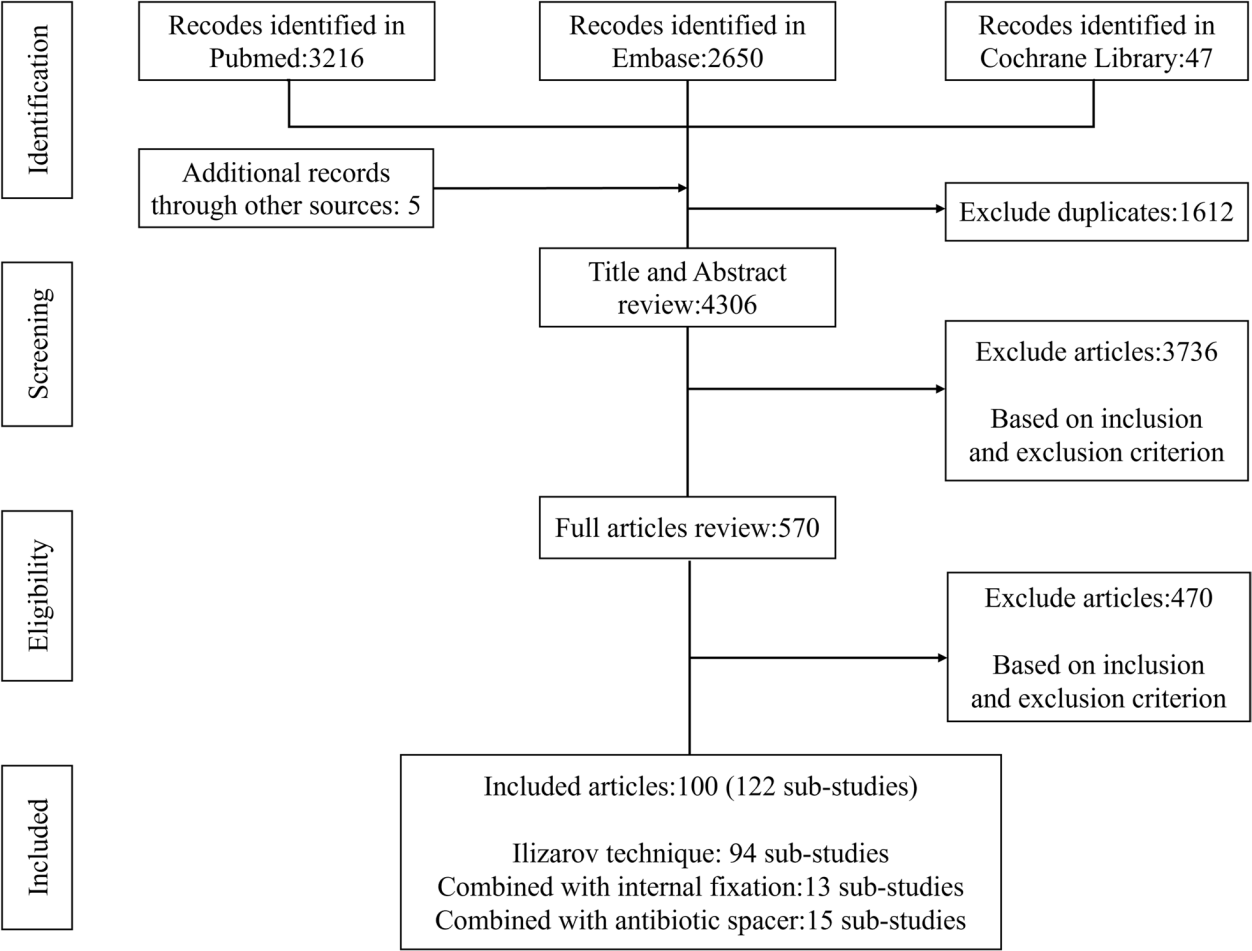
Flow chart of the literature review

The process for study selection was displayed in Fig. 1.

### Data extraction and study quality assessment

Two independent reviewers screened the titles and abstracts of initially identified articles according to the inclusion criteria. The potentially relevant articles then underwent full-text retrieval independently and repeatedly by two independent reviewers. Two investigators independently extracted data from the studies meeting the inclusion and exclusion criteria, after they achieved good agreement (kappa score = 0.93). Any differences between two reviewers were resolved by discussion. The following information were extracted: (1) basic study characteristics including first author name, published year, study design, and country of the study conducted. If the information of country was not mentioned in the article, the location of the first author’s or surgeon’s institution was extracted; (2) related patients baseline demographic data including number, mean age, male/female ratio, following up time; (3) related data of operative details including etiology of bone defects, mean bone defect size, time from injury to surgery, number of operations prior to current surgery, type of Ilizarov method, type of external frames, flap used or not; (4) outcome measures including bone union rate (defined as the proportion of patients who achieved successful bone healing), bone result or functional result according to ASAMI, EFT, EFI, rate of bone grafting used on the docking site, and complications related to the treatment (including pin infection, pin loosening, pain, refracture, limb discrepancy (> 2 cm), malalignment (> 5°), joint stiffness, recurrent infection, and amputation). In addition, we also extracted additional information of the Ilizarov method’s combined technique, for example, the ingredients of antibiotic spacer. The National Institutes of Health (NIH) quality assessment tool was used to evaluate the quality of studies included [28].

### Statistical analysis

Summary data were calculated by weighted means based on the sample size of each study, and was presented as proportions or numerical data. We combined effect size of interested outcomes for each study together by the inverse variance statistical method for the rate meta-analysis and used log transformation when needed. Pooled effect size was presented percentage with 95% confidence interval (CI), respectively. Heterogeneity and the proportion of variation between studies was quantified using the I2 statistic. I2 values below 50%, 50% ~ 75%, and above 75% was defined as low, moderate, and high heterogeneity, respectively. In consideration of the underlying differences between the included studies, we chose random-effects models to calculate combined effect size.

Then, we conducted the comparison of interested outcomes between conventional Ilizaorv method and its modified methods by using relative risk (RR) as effect measure. The similar comparisons were also conducted between Ilizarov methods and its combined methods.

 Data were analyzed with R 4.2.1. Two-sided *P*-value < 0.05 was considered statistically significant.

## Result

### The quality of studies included

Evaluation of study quality of included studies was presented in Additional file 1: Appendix 1.

### Ilizarov methods

The characteristics and details of treatment of studies were shown in Additional file 1: Appendix 2 to 4. Due to the huge differences between tibial and femoral bone defects, we presented and evaluated each interested outcome of two positional defects respectively. 47 case series studies (involving 48 sub-studies), 22 comparative studies (involving 30 sub-studies), and 3 randomized clinical trials (involving 5 sub-studies) were identified in tibial defects. 8 case series studies and 2 comparative studies (involving 3 sub-studies) were identified in femoral defects.

#### Ilizarov methods in different position

We presented the pooled effect size of union rate and specific complication incidence rate of Ilizarov method in Additional file 2. The pooled estimate of union rate in tibial and femoral defects were 99.29% (95% CI: 98.67% ~ 99.86%) and 98.81% (95% CI: 96.78% ~ 100.00%), respectively. We found significant difference that there was a degree of decrease of the incidence of pin loose (RR = 0.50, 95% CI: 0.31 ~ 0.83), pin infection (RR = 0.83, 95% CI: 0.74 ~ 0.92), malignment (> 5°) (RR = 0.43, 95% CI: 0.31 ~ 0.61) and joint stiffness (RR = 0.64 (95% CI: 0.51 ~ 0.80) in the tibia subgroup, compared to the femur subgroup. However, the findings of comparison of pin infection, malalignment and joint stiffness should be interpreted with caution for the moderate to high degree of heterogeneity.

#### Ilizarov methods by different types of external fixations

The pooled effect size and comparisons of each outcome of interest between conventional Ilizarov method and its modified method by type of external frame in tibial defects were shown in Table 1. The similar pooled estimation and comparisons were not conducted in the femoral defects for the limited number of original studies. The pooled union rate was 99.46% (95% CI: 98.82% ~ 100.00%) in ring frame method, and 98.37% (95% CI: 96.20% ~ 100.00%) in unilateral frame method. When further conducted the comparison of the relevant outcomes between two frame methods, the relative lower union rate was presented in the unilateral frame method (RR = 0.97, 95% CI: 0.94 ~ 1.00). We also found higher incidence rate of pain and malalignment when patients were applied with the unilateral external fixation (RR = 1.88, 95% CI: 1.11 ~ 3.16; RR = 2.50, 95% CI: 1.39 ~ 4.51, respectively), while with moderate to high degree of heterogeneity.

**Table 1:** Pooled estimation and comparative analysis of interested outcomes of Ilizarov method by different types of frame in tibial defects

	**Unilateral frame**			**Ring frame**			Comparison
	Studies (n) (Patients, n)	Pooled effect size (95% CI)	Heterogeneity (*I*^2^, %)	Studies (n) (Patients, n)	Pooled effect size (95% CI)	Heterogeneity (*I*^2^, %)	
Union rate	13 (257/272)	98.37 (96.20, 100.00)	6.7	57 (1549/1596)	99.46 (98.82, 100.00)	0.0	RR = 0.97 (0.94, 1.00)
Excellent rate in bone result	12 (140/247)	60.60 (50.63, 72.54)	76.0	36 (652/1130)	60.84 (54.70, 67.67)	79.0	RR = 0.98 (0.87, 1.11)
Pin infection	9 (77/139)	33.33 (19.10, 58.14)	85.6	40 (561/1092)	49.80 (41.43, 59.87)	90.4	RR = 1.08 (0.92, 1.27)
Pin loose	4 (8/98)	5.84 (0.79, 10.89)	13.8	9 (28/328)	7.38 (4.57, 10.19)	0.0	RR = 0.97 (0.45, 2.03)
Pain	3 (15/57)	21.93 (-21,22, 65.66)	95.6	9 (40/285)	10.82 (4.30, 17.35)	75.2	RR = 1.88 (1.11, 3.16)
Refracture^a^	6 (4/158)	1.07 (0.00, 3.58)	0.0	27 (35/786)	2.39 (1.10, 3.68)	0.0	RR = 0.57 (0.20, 1.58)
Limb discrepancy (> 2 cm)	3 (14/75)	18.45 (2.97, 33.94)	64.7	14 (29/389)	4.73 (2.15, 7.31)	33.9	RR = 2.50 (1.39, 4.51)
Malalignment (> 5°)	6 (15/138)	16.39 (10.79, 24.91)	12.7	24 (72/574)	17.02 (12.34, 23.47)	60.8	RR = 1.87 (0.51, 1.46)
Joint stiffness	7 (41/160)	25.71 (13.93, 37.48)	70.1	27 (150/658)	22.73 (13.10, 32.37)	97.5	RR = 1.12 (0.83, 1.51)
Recurrent infection	3 (2/82)	1.82 (0.00, 6.22)	10.4	36 (63/895)	1.97 (0.76, 3.18)	56.7	RR = 0.35 (0.09, 1.39)
Amputation	8 (2/162)	0.03 (0.00, 2.62)	0.0	45 (13/1225)	0.25 (0.00, 0.93)	0.0	RR = 1.16 (0.26, 5.11)

*RR* relative risk

^a^Either the regenerate bone or at the docking sit

#### Ilizarov methods by using flap or not

The pooled estimations and comparisons of outcomes of interest between conventional Ilizarov method and its modified method by the usage of flap in tibial defects were shown in the Table 2. The similar pooled estimation and comparisons were not conducted in the femoral defects for the limited number of original studies. We only found 8 studies that all included patients were asked to receive flaps during the surgery, and the pooled union rate was 98.26% (95% CI: 95.33% ~ 100.00%). When explored the impacts of applying flap, we did not find significant differences of interested outcomes between two groups. Unfortunately, the incidences of some interested complications were not compared between the two groups, due to the limited number of original studies using Ilizarov method with flap.

**Table 2:** Pooled estimation and comparative analysis of interested outcomes of Ilizarov method by the flap used or not in tibial defects

	**Applying flap with Ilizarov method**			**Conventional Ilizarov method**			
	Studies (n) (Patients, n)	Pooled effect size (95% CI)	Heterogeneity (*I*^2^, %)	Studies (n) (Patients, n)	Pooled estimate (%, 95% CI)	Heterogeneity (*I*^2^, %)	Comparison
Union rate	8 (159/170)	98.26 (95.33, 100.00)	27.7	42 (1099/1134)	99.53 (98.79, 100.00)	0.0	RR = 0.97 (0.92, 1.01)
Excellent rate in bone result	2 (16/34)	49.25 (34.83, 69.65)	0.0	31 (546/864)	67.15 (61.72, 73.06)	73.7	-
Pin infection	1 (5/18)	27.78 (13.19, 58.51)	-	33 (517/904)	56.57 (46.92, 68.20)	89.3	-
Pin loose	-	-	-	10 (232/325)	5.90 (3.36, 8.44)	0.0	-
Pain	-	-	-	8 (27/234)	8.75 (2.66, 14.84)	67.4	-
Limb discrepancy (> 2 cm)	9 (1/58)	5.13 (2.40, 7.86)	39.9	10 (30/344)	4.85 (1.17, 8.53)	56.1	RR = 0.20 (0.03, 1.43)
Malalignment (> 5°)	1 (0/166)	2.70 (0.18, 4.16)	-	19 (59/509)	16.72 (12.50, 22.35)	42.4	-
Joint stiffness	2 (8/58)	9.34 (0.00, 28.89)	86.6	22 (139/521)	25.31 (14.02, 36.61)	97.8	-
Recurrent infection	-	-	-	29 (51/710)	1.15 (0.06, 2.25)	61.9	-
Amputation	7 (3/126)	0.76 (0.00, 3.73)	0.0	30 (7/803)	0.15 (0.00, 0.98)	0.0	RR = 2.73 (0.72, 10.42)

*RR* relative risk

^a^Either the regenerate bone or at the docking site

### Combination of Ilizarov method with other techniques

#### Combined with antibiotic spacer and the comparison with Ilizarov methods

Fourteen studies (involving 15 sub-studies) were included in the final analysis, including 6 case series studies and 4 comparative studies (involving 5 sub-studies) of tibial defects and 4 case series studies of femoral defects. We presented the relevant information by tibial and femoral defects respectively, for the potential discrepancy between the two locations (Additional file 1: Appendix 5 to 7).

The summarized estimates of rate of union and specific complication incidence in tibial defects were shown in Table 3. The similar pooled estimations were not conducted in the femoral defects for the limited number of original studies. The pooled estimate of union rate was 99.58% (95% CI: 98.05% ~ 100.00%). For the limited number of studies with femoral defects, we only conducted the following comparisons between Ilizarov method and its combined method with antibiotic spacer in tibial defects (Table 3). We noticed the higher union rate (RR = 1.02, 95% CI: 1.01 ~ 1.04) and lower excellent rate in bone result (RR = 0.75, 95% CI: 0.61 ~ 0.91) in the combined group, compared to that in the Ilizarov method group.

**Table 3:** Pooled estimation and comparison of interested outcomes between Ilizarov method and its combined method in tibial defect

	**Combination with antibiotic spacer**			**Ilizarov method**			Comparison
	Studies (n) (Patients, n)	Pooled effect size (95% CI)	Heterogeneity (*I*^2^, %)	Studies (n) (Patients, n)	Pooled effect size (95% CI)	Heterogeneity (*I*^2^, %)	
Union rate	11 (274/279)	99.58 (98.05, 100.00)	0.0	83 (2069/2159)	99.29 (98.67, 99.86)	2.3	RR = 1.02 (1.01, 1.04)
Excellent rate in bone result	5 (58/136)	48.23 (40.15, 57.92)	46.9	54 (842/1472)	60.58 (55.78, 65.80)	76.3	RR = 0.75 (0.61, 0.91)
Pin infection	7 (93/193)	44.46 (24.46, 80.83)	92.3	54 (693/1363)	49.44 (42.01, 58.19)	89.5	RR = 0.95 (0.81, 1.11)
Pain	1 (2/32)	6.25 (0.00, 14.64)	-	12 (55/342)	13.48 (3.72, 23.24)	85.9	-
Refracture^a^	3 (4/65)	4.33 (0.00, 9.22)	0.0	37 (46/1089)	2.25 (1.16, 3.33)	0.0	RR = 1.46 (0.54, 3.92)
Limb discrepancy (> 2 cm)	1 (3/32)	9.38 (0.00, 19.47)	-	20 (46/590)	4.58 (2.55, 6.61)	45.1	-
Malalignment (> 5°)	2 (10/46)	16.28 (2.35, 100.00)	75.0	33 (93/768)	16.68 (12.87, 21.61)	54.3	-
Joint stiffness	5 (26/147)	20.94 (3.05, 38.83)	81.3	39 (222/974)	23.04 (15.82, 30.52)	96.0	RR = 0.78 (0.54, 1.12)
Recurrent infection	6 (11/146)	5.52 (0.00, 12.10)	59.3	43 (73/1098)	1.91 (0.85, 2.97)	52.4	RR = 1.13 (0.62, 2.09)
Amputation	11 (5/279)	0.42 (0.00, 1.95)	0.0	63 (19/1583)	0.28 (0.00, 0.91)	0.0	RR = 1.49 (0.56, 3.97)

*RR* relative risk

^a^Either the regenerate bone or at the docking site

#### Combined with internal fixation and the comparison with Ilizarov methods

Thirteen studies were included in the analysis. 2 case series studies and 4 comparative studies using Ilizarov method with intramedullary nailing and 3 case series studies using Ilizarov method with locking plate were identified in tibial defects; 2 case series studies and 2 comparative studies using Ilizarov method with intramedullary nailing were identified in femoral defects. In consideration of the potential differences between tibial and femoral defects and the different function of various internal fixations, we presented and evaluated the relevant outcomes by locations and types of internal fixations (Additional file 1: Appendix 8 to 10).

The pooled union rate and summarized estimates of rate of specific complication incidence in tibial defects was shown in Table 4. The pooled union rate of Ilizarov method combined with intramedullary nailing was 95.02% (95% CI: 87.28% ~ 100.00%). Table 4 showed the result of comparisons between Ilizarov method and Ilizarov method combined with intramedullary nailing, while, the similar comparisons were not conducted between other groups for the limited number of original studies. Compared to Ilizarov method, lower incidence of pin infection was observed in combined group (RR = 0.17, 95% CI: 0.07 ~ 0.46), with high degree of statistical heterogeneity. In addition, we found the higher incidence of recurrent infection (RR = 2.22, 95% CI: 1.17 ~ 4.22) and amputation (RR = 6.48, 95%CI: 2.78 ~ 15.01) in combined group. However, due to the presence of high degree of statistical heterogeneity, this finding about the comparison of recurrent infection between the two groups should be interpreted with caution. The similar pooled estimations and comparisons were not conducted in the femoral defects for the limited number of original studies.

**Table 4:** Pooled estimation and comparison of interested outcomes between Ilizarov methods and its combined method in tibial defect

	**Combination with intramedullary nailing**			**Ilizarov method**			Comparison
	Studies (n) (Patients, n)	Pooled estimate (%,95% CI)	Heterogeneity (*I*^2^, %)	Studies (n) (Patients, n)	Pooled estimate (%,95% CI)	Heterogeneity (*I*^2^, %)	
Union rate	6 (81/90)	95.02 (87.28, 100.00)	58.4	83 (2069/2159)	99.29 (98.67, 99.86)	2.3	RR = 0.94 (0.88, 1.01)
Excellent rate in bone result	5 (37/58)	65.92 (54.68, 79.48)	0.0	54 (842/1472)	60.58 (55.78, 65.80)	76.3	RR = 1.12 (0.91, 1.36)
Pin infection	3 (4/44)	7.89 (0.00, 15.80)	0.0	54 (693/1363)	49.44 (42.01, 58.19)	89.5	RR = 0.17 (0.07, 0.46)
Pain	-	-	-	12 (55/342)	13.48 (3.72, 23.24)	85.9	-
Refracture^a^	-	-	-	37 (46/1089)	2.25 (1.16, 3.33)	0.0	-
Limb discrepancy (> 2 cm)	-	-	-	20 (46/590)	4.58 (2.55, 6.61)	45.1	-
Malalignment	3 (4/47)	7.52 (0.03, 15.02)	0.0	33 (93/768)	16.68 (12.87, 21.61)	54.3	RR = 0.70 (0.27, 1.83)
Joint stiffness	1 (1/11)	7.14 (0.00, 20.63)	-	39 (222/974)	23.04 (15.82, 30.52)	96.0	-
Recurrent infection	4 (9/61)	13.08 (0.00, 27.90)	71.6	43 (73/1098)	1.91 (0.85, 2.97)	52.4	RR = 2.22 (1.17, 4.22)
Amputation	6 (7/90)	1.82 (0.00, 5.93)	39.7	63 (19/1583)	0.28 (0.00, 0.91)	0.0	RR = 6.48 (2.78, 15.01)

*RR* relative risk

^a^Either the regenerate bone or at the docking site

## Discussion

We reviewed the studies focused on the long bone defects of low extremity treated by Ilizarov methods and its combined technique. An evidence-based summary was synthesized and supported the effectiveness of Ilizarov methods and its combined technique in the management of bone defects for the desired union rate. In addition, our study supported that combined technique had lower complications than Ilizarov methods. However, the findings need to be confirmed by further studies. This review provides valuable insights into the effects and limitations of various Ilizarov methods, enabling researchers to assess emerging trends and potentially promote ongoing innovation in this field. Additionally, this review may present multifaceted solutions for clinicians and patients in various clinical practices, aiming to enhance the treatment outcomes.

The first systematic review demonstrating the capability of Ilizarov technique to treat bone defect of lower extremities was conducted by Papakostidis et al. [26] in 2013, with 37 articles included by retrieving from Medline electronic database only. This article reported that pooled estimations of union rate were 94.3% and 96.4% in tibial and femoral group respectively, which was a little bit lower than those in our study (99.29% and 98.81%, respectively). Apart from the improvement of the technique system over time, we noticed that the report conducted by Papakostidis et al. took articles with no bone defect patients into consideration, which may lead to potential differences. And then, Yin et al. published two reports focused on infective tibial defects in 2014 [29] and infective tibial and femoral defects in 2015 [25], respectively. The mean union rate reported in two studies (nearly 96%) was similar to that in our study.

In our result, unilateral frame fixator systems achieved the desired union rate. While, lower union rate was shown in unilateral frame group compared to that in ring frame group, which seems caused by the less stability of unilateral frame. We only found two clinical trials focused on comparing effectiveness of ring and unilateral fixation systems [30, 31]. The one of article reported that the unilateral frame was related with more residual problems, when bone gap was more than 6 cm [30]. The only significant difference in another article was that the group with unilateral frame had better radiological quality of regenerate in patients with bone defect measuring 7 cm or less [31]. Thus, the application of unilateral fixation seems more likely to achieve better bone result in patients with small bone gap, and further studies with larger sample sizes should be conducted to explore the contention.

We only retrieved 8 studies including more than 10 patients applied for Ilizarov method with flap, and did not find any significant differences between Ilizarov method with flap and conventional Ilizarov method in the current studies identified. Unfortunately, comparison was not made between different types of flaps for the limited number of original studies. Given the success of the modified Ilizarov method requires well-coordinated team of plastic and orthopedic surgeons, key influential factors and more details of the technique such as the timing of tissue coverage should be explored in the further studies.

Our result reported desired union rate of Ilizaorv methods with antibiotic spacer. Ren et al. reported the meta-analysis included eleven studies written in Chinese and English in 2021, which focused on synthesizing the excellent rate in bone results and reinfection rate in the management on infective tibial bone defects by the combined technique [27]. This article showed the similar result of our study for low pooled reinfection incidence rate (6.99%, 95% CI: 5.20% ~ 32.50% vs 6.03%, 95% CI: 0.00% ~ 15.87%). While higher pooled excellent rate in bone result was observed in the report conducted by Ren et al. than that in our study (65%, 95% CI: 22.0% ~ 97.0% vs 41.94%, 95% CI: 29.20% ~ 60.24%), which may be associated with the different characteristic of included studies. In addition, we further pooled rate of specific complication incidence and conducted comparisons of outcomes of interest between Ilizarov method and its combination with antibiotic spacer. The pooled estimate of effect size showed slightly higher union rate in the combined technique group. Although several findings supported the fact that antibiotic carrier systems are capable of releasing antibiotics directly at the affected or targeted area, leading to higher antibiotic concentrations than those achieved through systemic administration [32], and this localized antibiotic delivery has been demonstrated to be effective in eradicating infections and reducing the risk of recurrence [33]. However, our findings did not observe the significant difference in recurrence infection rate between Ilizarov methods and its combined technique with antibiotic spacer. Additionally, we noticed polymethylmethacrylate (PMMA) was used most frequently among the included articles of our study. While, for no need of additional surgeons to take out, an increasing number of surgeons prefer to apply local biodegradable carriers to treat the dead space in recent years. Only 2 studies were identified in the last two years applied biodegradable system (made of antibiotics with calcium sulfate) with Ilizarov method for the management of bone loss [34, 35]. However, high quality clinical evidence of this combined technique does not exist.

To our acknowledge, there were not relevant systematic review published previously focused on Ilizarov method combined with internal fixation in the treatment of bone defects. Our result showed the desired union rate in Ilizarov method combined with internal fixation. The significantly lower pin infection observed in combined group, with high degree of statistical heterogeneity. In addition, we also noticed that patients received the combined technique of Ilizarov method with intramedullary nailing showed shorter EFI than those with only Ilizaorv method either in tibial and femoral defects (0.6, range: 0.5–0.9 vs 1.6, range: 0.7–3.0 in tibial defects and 1.5, range: 0.4–4.4 vs 2.1, range: 0.9–4.4 in femoral defects). The shorter EFT may be attributed to the contribution of overall strength and stability provided from internal fixation [22]. Complications during treatment are often associated with the duration of external fixation, thus, an ideal limb reconstruction strategy aims to minimize the duration of external fixation to enable earlier rehabilitation for patients [36]. However, it's essential to acknowledge that the combination of internal fixation with the Ilizarov method, while reducing complications and EFT, also carries its own risks, such as implant failure and infection [37]. As reported in our study, although with high degree of heterogeneity, the higher incidence rate of recurrent infection and amputation were observed in the combined group of Ilizarov method with intramedullary nailing, compared to Ilizarov method. In addition, we also noticed that Ilizarov method with intramedullary nailing in combination with antibiotic carriers may also play a valid role in managing infection-induced large bone defects and eliminating infection effectively [38], which was conducted by Xu et.al in 2022. More studies will be required to explore the effectiveness of the combined technique of Ilizarov method with internal fixations to inform practice.

Although this study comprehensively and systematically evaluated the efficacy of the Ilizarov and its combined methods, there are still some limitations should be considered. Due to the number of randomized controlled trials or high-quality studies were not powerful for conducting systematic review, the current review was still based on data mostly extracted from case series. Moreover, there are disparity in the number of studies available for the Ilizarov methods compared to the number studies concerning the combined methods, which may impact the depth and breadth of our analysis and influence the generalizability of the results. Besides, high heterogeneity existed in most pooling data in our study, which may result from different research quality, various surgeons’ experience and diversity of rehabilitation nursing. While, the more important point was that there was lack of homogenous criteria in documenting. For example, some studies documented the specific number of pin track, while other documented the number of patients occurred pin infection. Besides, a lot of items were not reported. Although we tried our best to ensure the analysis exactly by excluding the data for not documenting accurately, the number of available data for analysis decreased greatly and limited some evaluations unfortunately. Thus, there is a need to improve the harmonization of the collection of data relating to the outcome of treatment, and more large-size prospective studies and comparative researches should be conducted in the future.

## Conclusion

In summary, our research supported the reliable treatment of Ilizarov methods in the bone defects. Furthermore, our findings revealed that combining the Ilizarov techniques with other technique may be an applicable choice for reconstructing the bone defects. However, it's important to note that compared to Ilizarov methods, higher rate of some complications was observed in the combined technique, and further studies are needed to confirm these findings.

### Supplementary Information

**Additional file 1: Appendix 1.** Evaluation of included studies using the National Institutes of Health quality assessment tool. **Appendix 2.** Descriptive characteristics of included studies (Ilizarov method without combinations). **Appendix 3.** Details of Ilizarov method without combinations. **Appendix 4.** Interested outcomes of included studies (ilizarov method without combinations). **Appendix 5.** Descriptive characteristics of included studies (Ilizarov technique combined with antibiotic spacer). **Appendix 6.** Details of Ilizarov technique combined with antibiotic spacer. **Appendix 7.** Interested outcomes of included studies (Ilizarov technique combined with antibiotic spacer). **Appendix 8.** Descriptive characteristics of included studies (Ilizarov technique combined with internal fixation). **Appendix 9.** Details of Ilizarov technique combined with internal fixation. **Appendix 10.** Outcomes of included studies (Ilizarov technique combined with internal fixation).

** Additional file 2.** Pooled estimation and comparative analysis of interested outcomes of Ilizarov method by locations.

## Data Availability

The data of this study are included in the manuscript and supplementary materials.

## Abbreviations

EFT: Long external fixation time
ASAMI: Association for the Study of the Method of Ilizarov
EFI: External fixation index
CI: Confidence interval
RR: Relative risk
PMMA: Polymethylmethacrylate
