# Supplementary material for: Ilizarov method and its combined methods in the treatment of long bone defects of the lower extremity: systematic review and meta-analysis
Source: BMC Musculoskelet Disord. 2023 Nov 16;24:891. doi: 10.1186/s12891-023-07001-9 (PMC10652567; doi:10.1186/s12891-023-07001-9)
Supplement: Supplementary file 1 — Additional file 1: Appendix 1. Evaluation of included studies using the National Institutes of Health quality assessment tool. Appendix 2. Descriptive characteristics of included studies (Ilizarov method without combinations). Appendix 3. Details of Ilizarov method without combinations. Appendix 4. Interested outcomes of included studies (ilizarov method without combinations). Appendix 5. Descriptive characteristics of included studies (Ilizarov technique combined with antibiotic spacer). Appendix 6. Details of Ilizarov technique combined with antibiotic spacer. Appendix 7. Interested outcomes of included studies (Ilizarov technique combined with antibiotic spacer). Appendix 8. Descriptive characteristics of included studies (Ilizarov technique combined with internal fixation). Appendix 9. Details of Ilizarov technique combined with internal fixation. Appendix 10. Outcomes of included studies (Ilizarov technique combined with internal fixation). [file 12891_2023_7001_MOESM1_ESM.docx]

**Appendix 1.** Evaluation of included studies using the National Institutes of Health quality assessment tool.

| Studies | Objective clearly? | Inclusive criteria descripted? | Participants representative? | Patients met the criteria? | Sample enough? | Intervention clearly described? | Outcome well defined? | Blinded? | Loss to follow-up  < 20%？ | Statistical analysis done? | Multiple outcome measures? | Group-level intervention? |
| --- | --- | --- | --- | --- | --- | --- | --- | --- | --- | --- | --- | --- |
| Paley[1] | Yes | No | Yes | No | Yes | Yes | Yes | No | Yes | No | No | NA |
| Cattaneo[2] | Yes | No | Yes | No | Yes | Yes | Yes | No | Yes | No | No | NA |
| Cierny[3] | Yes | No | Yes | No | Yes | Yes | No | No | Yes | No | No | NA |
| Marsh[4] | Yes | No | Yes | No | Yes | Yes | No | No | Yes | No | No | NA |
| Hosny[5] | Yes | No | Yes | No | Yes | Yes | Yes | No | Yes | No | No | NA |
| Ring[6] | Yes | No | Yes | No | Yes | Yes | No | No | Yes | No | No | NA |
| Paley[7] | Yes | No | Yes | No | Yes | Yes | Yes | No | Yes | No | No | NA |
| Maini[8] | Yes | No | Yes | No | Yes | Yes | Yes | No | Yes | No | No | NA |
| Atesalp[9] | Yes | No | Yes | No | Yes | Yes | Yes | No | Yes | No | No | NA |
| Bobroff[10] | Yes | No | Yes | No | Yes | Yes | Yes | No | Yes | No | No | NA |
| Sen[11] | Yes | No | Yes | No | Yes | Yes | Yes | No | Yes | No | No | NA |
| Mekhail[12] | Yes | No | Yes | No | Yes | Yes | Yes | No | Yes | Yes | No | NA |
| Mahaluxmivala  [13] | Yes | No | Yes | No | Yes | Yes | No | No | Yes | No | No | NA |
| Rozbruch[14] | Yes | No | Yes | No | Yes | Yes | Yes | No | Yes | No | No | NA |
| Baruah[15] | Yes | No | Yes | No | Yes | Yes | Yes | No | Yes | No | No | NA |
| Emara[16] | Yes | Yes | Yes | Yes | Yes | Yes | Yes | No | Yes | No | No | NA |
| Madhusudhan  [17] | Yes | No | Yes | No | Yes | Yes | Yes | No | Yes | No | No | NA |
| Pirwani[18] | Yes | Yes | Yes | Yes | Yes | Yes | Yes | No | Yes | - | No | NA |
| Bumbasirević  [19] | Yes | No | Yes | No | Yes | Yes | Yes | No | Yes | No | No | NA |
| Wu[20] | Yes | No | Yes | No | Yes | Yes | Yes | No | Yes | Yes | No | NA |
| Liodakis[21] | Yes | Yes | Yes | Yes | Yes | Yes | Yes | No | Yes | Yes | No | NA |
| Chim[22] | Yes | No | Yes | No | Yes | Yes | No | No | Yes | No | No | NA |
| Lin[23] | Yes | No | Yes | No | Yes | Yes | No | No | Yes | No | No | NA |
| Babar[24] | - | - | - | - | Yes | - | - | - | - | - | No | NA |
| Sala[25] | Yes | Yes | Yes | Yes | Yes | Yes | Yes | No | Yes | Yes | No | NA |
| Xu[26] | Yes | No | Yes | No | Yes | Yes | Yes | No | Yes | No | No | NA |
| Feng[27] | - | - | - | - | Yes | - | - | - | - | - | No | NA |
| Krappinger[28] | Yes | No | Yes | No | Yes | Yes | Yes | No | Yes | No | No | NA |
| Selim[29] | Yes | Yes | Yes | Yes | Yes | Yes | Yes | No | Yes | No | No | NA |
| Atef[30] | Yes | Yes | Yes | Yes | Yes | Yes | Yes | No | Yes | No | No | NA |
| Yin[31] | Yes | Yes | Yes | Yes | Yes | Yes | Yes | No | Yes | No | No | NA |
| Morsy[32] | - | - | - | - | Yes | - | - | - | - | - | No | NA |
| Yin[33] | Yes | Yes | Yes | Yes | Yes | Yes | Yes | No | Yes | No | No | NA |
| Ajmera[34] | Yes | Yes | Yes | Yes | Yes | Yes | Yes | No | Yes | No | No | NA |
| Azzam[35] | Yes | No | Yes | No | Yes | Yes | Yes | No | Yes | No | No | NA |
| Bernstein[36] | Yes | No | Yes | No | Yes | Yes | Yes | No | Yes | Yes | No | NA |
| Fürmetz[37] | Yes | Yes | Yes | Yes | Yes | Yes | Yes | No | Yes | No | No | NA |
| Rohilla[38] | Yes | Yes | Yes | Yes | Yes | Yes | Yes | No | Yes | Yes | No | NA |
| Sadek[39] | Yes | No | Yes | No | Yes | Yes | Yes | No | Yes | Yes | No | NA |
| Meleppuram[40] | Yes | Yes | Yes | Yes | Yes | Yes | Yes | No | Yes | No | No | NA |
| Tesworth[41] | Yes | Yes | Yes | Yes | Yes | Yes | Yes | No | Yes | Yes | No | NA |
| Wang[42] | Yes | Yes | Yes | Yes | Yes | Yes | Yes | No | Yes | Yes | No | NA |
| Xu[43] | Yes | Yes | Yes | Yes | Yes | Yes | No | No | Yes | No | No | NA |
| Zhang[44] | Yes | Yes | Yes | Yes | Yes | Yes | Yes | No | Yes | No | No | NA |
| Wu[45] | Yes | No | Yes | No | Yes | Yes | Yes | No | Yes | Yes | No | NA |
| Catagni[46] | Yes | Yes | Yes | Yes | Yes | Yes | Yes | No | Yes | Yes | No | NA |
| Rohilla[47] | Yes | Yes | Yes | Yes | Yes | Yes | Yes | No | Yes | Yes | No | NA |
| Fahad[48] | Yes | Yes | Yes | Yes | Yes | Yes | Yes | No | Yes | No | No | NA |
| Yikemu[49] | Yes | Yes | Yes | Yes | Yes | Yes | Yes | No | Yes | Yes | No | NA |
| Bakhsh[50] | Yes | Yes | Yes | Yes | Yes | Yes | Yes | No | Yes | No | No | NA |
| Kinik[51] | Yes | Yes | Yes | Yes | Yes | Yes | Yes | No | Yes | No | No | NA |
| Jilani[52] | Yes | Yes | Yes | Yes | Yes | Yes | Yes | No | Yes | No | No | NA |
| Li[53] | Yes | Yes | Yes | Yes | Yes | Yes | Yes | No | Yes | Yes | No | NA |
| Li[54] | Yes | Yes | Yes | Yes | Yes | Yes | Yes | No | Yes | No | No | NA |
| Wadhwani[55] | Yes | Yes | Yes | Yes | Yes | Yes | Yes | No | Yes | Yes | No | NA |
| Brauns[56] | Yes | No | Yes | No | Yes | Yes | Yes | No | Yes | No | No | NA |
| Abdou[57] | Yes | Yes | Yes | Yes | Yes | Yes | Yes | No | Yes | No | No | NA |
| Abula[58] | Yes | No | Yes | No | Yes | Yes | Yes | No | Yes | No | No | NA |
| Baruah[59] | Yes | Yes | Yes | Yes | Yes | Yes | No | No | Yes | Yes | No | NA |
| Li[60] | Yes | No | Yes | No | Yes | Yes | Yes | No | Yes | No | No | NA |
| Xu[61] | Yes | Yes | Yes | Yes | Yes | Yes | Yes | No | Yes | No | No | NA |
| Xiayimaierdan  [62] | Yes | Yes | Yes | Yes | Yes | Yes | Yes | No | Yes | Yes | No | NA |
| Huang[63] | Yes | Yes | Yes | Yes | Yes | Yes | Yes | No | Yes | Yes | No | NA |
| Huang[64] | Yes | Yes | Yes | Yes | Yes | Yes | Yes | No | Yes | Yes | No | NA |
| Rollo[65] | Yes | No | Yes | No | Yes | Yes | Yes | No | Yes | Yes | No | NA |
| Kliushin[66] | Yes | No | Yes | No | Yes | Yes | Yes | No | Yes | No | No | NA |
| Hamiti[67] | Yes | Yes | Yes | Yes | Yes | Yes | Yes | No | Yes | Yes | No | NA |
| Rohilla[68] | Yes | Yes | Yes | Yes | Yes | Yes | Yes | No | Yes | Yes | No | NA |
| Arfee[69] | Yes | No | Yes | No | Yes | Yes | Yes | No | Yes | Yes | No | NA |
| Yushan[70] | Yes | Yes | Yes | Yes | Yes | Yes | Yes | No | Yes | No | No | NA |
| Abulaiti[71] | Yes | Yes | Yes | Yes | Yes | Yes | Yes | No | Yes | Yes | No | NA |
| Krishnan[72] | Yes | No | Yes | No | Yes | Yes | Yes | No | Yes | No | No | NA |
| Saridis[73] | Yes | Yes | Yes | Yes | Yes | Yes | Yes | No | Yes | No | No | NA |
| Arora[74] | Yes | No | Yes | No | Yes | Yes | Yes | No | Yes | No | No | NA |
| Wan[75] | Yes | Yes | Yes | Yes | Yes | Yes | Yes | No | Yes | Yes | No | NA |
| Agrawal[76] | Yes | No | Yes | No | Yes | Yes | Yes | No | Yes | No | No | NA |
| Liu[77] | Yes | No | Yes | No | Yes | Yes | Yes | No | Yes | Yes | No | NA |
| Sen[78] | Yes | Yes | Yes | Yes | Yes | Yes | Yes | No | Yes | Yes | No | NA |
| Bakhsh[79] | Yes | Yes | Yes | Yes | Yes | Yes | Yes | No | Yes | No | No | NA |
| Rohilla[80] | Yes | Yes | Yes | Yes | Yes | Yes | Yes | No | Yes | Yes | No | NA |
| Hutson Jr[81] | Yes | No | Yes | No | Yes | Yes | No | No | Yes | No | No | NA |
| Spiegl[82] | Yes | Yes | Yes | Yes | Yes | Yes | Yes | No | Yes | Yes | No | NA |
| Peng[83] | Yes | No | Yes | No | Yes | Yes | Yes | No | Yes | No | No | NA |
| van Niekerk[84] | Yes | Yes | Yes | Yes | Yes | Yes | Yes | No | Yes | Yes | No | NA |
| Hamiti[85] | Yes | Yes | Yes | Yes | Yes | Yes | Yes | No | Yes | No | No | NA |
| Liu[86] | Yes | Yes | Yes | Yes | Yes | Yes | Yes | No | Yes | Yes | No | NA |
| Khaled[87] | Yes | Yes | Yes | Yes | Yes | Yes | Yes | No | Yes | No | No | NA |
| Xu[88] | Yes | Yes | Yes | Yes | Yes | Yes | Yes | No | Yes | Yes | No | NA |
| Song[89] | Yes | Yes | Yes | Yes | Yes | Yes | Yes | No | Yes | No | No | NA |
| Sen[90] | Yes | Yes | Yes | Yes | Yes | Yes | Yes | No | Yes | No | No | NA |
| Peng[91] | Yes | Yes | Yes | Yes | Yes | Yes | Yes | No | Yes | Yes | No | NA |
| Oh[92] | Yes | Yes | Yes | Yes | Yes | Yes | Yes | No | Yes | No | No | NA |
| Liodakis[93] | Yes | Yes | Yes | Yes | Yes | Yes | Yes | No | Yes | Yes | No | NA |
| Eralp[94] | Yes | No | Yes | No | Yes | Yes | Yes | No | Yes | Yes | No | NA |
| Bas[95] | Yes | Yes | Yes | Yes | Yes | Yes | Yes | No | Yes | Yes | No | NA |
| Oh[96] | Yes | Yes | Yes | Yes | Yes | Yes | Yes | No | Yes | No | No | NA |
| Gupta[97] | Yes | Yes | Yes | Yes | Yes | Yes | Yes | No | Yes | No | No | NA |
| Lu[98] | Yes | No | Yes | No | Yes | Yes | Yes | No | Yes | No | No | NA |
| Li[99] | Yes | No | Yes | No | Yes | Yes | Yes | No | Yes | No | No | NA |
| Borzunov  [100] | Yes | Yes | Yes | Yes | Yes | Yes | Yes | No | Yes | Yes | No | NA |

Objective clearly?: Was the study question or objective clearly stated?

Inclusive criteria descripted?: Were eligibility/selection criteria for the study population prespecified and clearly described?

Participants representative?: Were the participants in the study representative of those who would be eligible for the test/service/intervention in the general or clinical population of interest?

Patients met the criteria?: Were all eligible participants that met the prespecified entry criteria enrolled?

Sample enough?: Was the sample size sufficiently large to provide confidence in the findings?

Intervention clearly described?: Was the test/service/intervention clearly described and delivered consistently across the study population?

Outcome well defined?: Were the outcome measures prespecified, clearly defined, valid, reliable, and assessed consistently across all study participants?

Blinded?: Were the people assessing the outcomes blinded to the participants' exposures/interventions?

Loss to follow-up < 20%？: Was the loss to follow-up after baseline 20% or less? Were those lost to follow-up accounted for in the analysis?

Statistical analysis done?: Did the statistical methods examine changes in outcome measures from before to after the intervention? Were statistical tests done that provided p values for the pre-to-post changes?

Multiple outcome measures?: Were outcome measures of interest taken multiple times before the intervention and multiple times after the intervention (i.e., did they use an interrupted time-series design)?

Group-level intervention?: If the intervention was conducted at a group level (e.g., a whole hospital, a community, etc.) did the statistical analysis take into account the use of individual-level data to determine effects at the group level?

**Appendix 2.** Descriptive characteristics of included studies (Ilizarov method without combinations)

| Author | Country | Published  year | Design | Treatment  period | Number | Age  (range) | Male/  female | Mean follow-up time (mths) (range/SD) | Follow-up rate (%) |
| --- | --- | --- | --- | --- | --- | --- | --- | --- | --- |
| **Tibia** | | | | | | | | | |
| Paley[1] | USA | 1989 | RS | 1982-1989 | 25 | 34(19-62) | 19/6 | nr | 100 |
| Cattaneo[2] | Italy | 1992 | RS | nr | 28 | 34(17-58) | 28/0 | nr | 100 |
| Cierny[3] | USA | 1994 | PC | 1988 to nr | 21 | nr | nr | nr(>24) | 100 |
| Marsh[4] | USA | 1994 | RS | 1989-1990 | 10 | 31(nr) | 5/5 | nr | 100 |
| Hosny[5] | Egypt | 1998 | RS | nr | 11 | 27(17-51) | 8/3 | 13(10-24) | 100 |
| Ring[6] | USA | 1999 | RC | 1983-1989 | 10 | 34(21-54) | 6/4 | 72(36-84) | 100 |
| Paley[7] | USA | 2000 | RS | 1987-1991 | 19 | 38(20-66) | 14/5 | 78(60-108) | 100 |
| Maini[8] | India | 2000 | RS | nr | 23 | 33(17-60) | 12/2 | 32(19-49) | 100 |
| Atesalp[9] | Turkey | 2002 | RS | 1995-2001 | 14 | 25(19-45) | 11/3 | 33(12-60) | nr |
| Bobroff[10] | USA | 2003 | RS | 1990-1999 | 12 | 32(20-50) | 12/0 | 15(3-48) | 100 |
| Sen[11] | Turkey | 2004 | RS | 1997-1999 | 24 | 31(18-53) | 18/6 | 30(18-60) | 100 |
| Mekhail[12] | USA | 2004 | RS | 1989-2001 | 15 | 38(20-70) | 11/4 | nr | 100 |
| Mahaluxmivala  [13] | UK | 2005 | RS | 1995-2001 | 12 | 38(26-52) | 11/1 | nr(>18) | 100 |
| Rozbruch[14] | USA | 2006 | RS | 1996-2004 | 25 | 34(16-61) | 17/8 | 36(13-72) | 100 |
| Baruah[15] | India | 2007 | RC | 1994-2003 | 25 | nr(18-50) | 22/3 | nr(24-72) | nr |
| Baruah[15] | India | 2007 | RC | 1994-2003 | 25 | nr(18-50) | 23/2 | nr(24-72) | nr |
| Emara[16] | Egypt | 2008 | PC | 2000-2001 | 16 | nr | nr | 36(22-48) | 100 |
| Madhusudhan  [17] | UK | 2008 | PC | nr | 22 | 37(20-52) | 18/4 | 13(6-20) | 92.3 |
| Pirwani[18] | Pakistan | 2008 | RS | 2004-2006 | 16 | 32(20-60) | 16/0 | 13(6-20) | 100 |
| Bumbasirević  [19] | Serbia | 2010 | RS | 1991-1996 | 30 | 30(20-49) | 29/1 | 99(62-122) | 100 |
| Wu[20] | China | 2011 | RS | 2000-2006 | 25 | 33(22-65) | 20/5 | 38(24-54) | 88.0 |
| Liodakis[21] | Germany | 2011 | RC | 1992-2007 | 21 | 47(15) | 16/5 | 95(67)* | 90.5 |
| Chim[22] | USA | 2011 | RS | 2000-2008 | 28 | 45(21-68) | 21/7 | 49(12-102) | 100 |
| Lin[23] | China | 2012 | RS | 1997-2012 | 16 | 36(18-70) | nr | nr | 100 |
| Babar[24] | India | 2013 | PC | 2009-2011 | 17 | 33(18-52) | 15/2 | nr | 100 |
| Sala[25] | Italy | 2013 | RC | 2003-2009 | 18 | 38(19-67) | 13/5 | 32(20-48) | 100 |
| Xu[26] | China | 2013 | RS | 2003-2011 | 30 | 34(19-49) | 21/9 | 29(12-72) | 100 |
| Feng[27] | China | 2013 | RS | nr | 21 | 35(19-49) | 15/6 | 31(12-72) | 90.4 |
| Krappinger[28] | Austria | 2013 | PS | 2004-2009 | 15 | 32(16-61) | 11/4 | 17(nr) | 100 |
| Selim[29] | Egypt | 2013 | RC | 2010-2011 | 10 | 30(22-40) | 10/0 | 29(nr) | 100 |
| Atef[30] | Egypt | 2014 | RS | 2007-2009 | 28 | 32(20-55) | 20/8 | nr(>12) | 100 |
| Yin[31] | China | 2014 | RS | 2004-2011 | 66 | 37(18-62) | 62/4 | 26(18-46) | 90 |
| Morsy[32] | Egypt | 2014 | PC | 2010-2013 | 12 | 37(24-48) | 10/2 | 9(6-20) | 100 |
| Yin[33] | China | 2015 | RS | 2004-2013 | 72 | 39*(18-62) | 60/12* | 23(14-46)* | 90.3 |
| Ajmera[34] | India | 2015 | RS | 2009-2012 | 30 | 33(20-48) | 23/2 | 15(nr) | 83.3 |
| Azzam[35] | Egypt | 2015 | RS | 2011-2013 | 30 | 32(18-52) | 30/0 | 18(10-32) | 100 |
| Bernstein[36] | USA | 2015 | RC | 2006-2012 | 30 | 43(25-56) | 24/6 | 31(nr) | 100 |
| Fürmetz[37] | Germany | 2016 | RS | 2000-2010 | 25 | 42(21-65) | 20/5 | 46(nr) | 100 |
| Rohilla[38] | India | 2016 | RCT | 2008-2013 | 35 | 33(18-64) | 32/3 | 34(nr) | nr |
| Rohilla[38] | India | 2016 | RCT | 2008-2013 | 35 | 30(18-65) | 30/5 | 33(nr) | nr |
| Sadek[39] | Egypt | 2016 | RC | nr | 14 | nr(17-51) | 12/2 | nr | nr |
| Meleppuram[40] | India | 2016 | RS | 2012-2015 | 42 | 38(26-64) | 32/10 | 14(10-24) | nr |
| Tesworth[41] | USA | 2017 | RS | nr | 21 | 38(18-66) | 18/3 | 31(12-84) | 100 |
| Wang[42] | China | 2017 | RS | 2012-2014 | 15 | 37(20-55) | 12/3 | nr(>24) | 100 |
| Xu[43] | China | 2017 | RS | 2007-2012 | 18 | 41(28-52) | 13/5 | 38(25-52) | 100 |
| Zhang[44] | China | 2018 | RS | 2010-2015 | 16 | 39(16-65) | 9/7 | 30(nr) | 100 |
| Wu[45] | China | 2018 | RC | 2007-2016 | 23 | 39(16-67) | 15/8 | 29(18-54) | 100 |
| Wu[45] | China | 2018 | RC | 2007-2016 | 17 | 39(18-65) | 12/5 | 29(18-54) | 100 |
| Catagni[46] | Italy | 2019 | RC | 2008-2015 | 45 | 43*(23-54) | 39/6 | nr | nr |
| Catagni[46] | Italy | 2019 | RC | 2008-2015 | 41 | 42*(33-52) | 38/3 | nr | nr |
| Rohilla[47] | India | 2019 | RCT | 2011-2016 | 15 | 30(18-50) | nr | 22(nr) | 100 |
| Rohilla[47] | India | 2019 | RCT | 2011-2016 | 15 | 35(18-60) | nr | 22(nr) | 100 |
| Fahad[48] | Pakistan | 2019 | RS | 2005-2016 | 51 | 46(16-69) | 41/10 | 37(nr) | 100 |
| Yikemu[49] | China | 2019 | RS | 2015-2017 | 78 | 45(23-68) | 52/26 | 19(23-36) | 100 |
| Bakhsh[50] | Pakistan | 2019 | RS | 2015-2017 | 56 | 33(16-50) | 53/3 | 20(7-36) | 100 |
| Kinik[51] | Turkey | 2019 | RS | 2000-2017 | 30 | 40(16-68) | 28/2 | 33(12-72) | 100 |
| Jilani[52] | India | 2020 | PS | 2014-2016 | 22 | 31(16-55) | 17/5 | 11(nr) | 100 |
| Li[53] | China | 2020 | RC | 2010-2017 | 13 | 41(30-56) | 10/3 | 29(nr)* | 100 |
| Li[53] | China | 2020 | RC | 2010-2017 | 13 | 40(22-54) | 10/3 | 29(nr)* | 100 |
| Li[54] | China | 2020 | RS | 2014-2017 | 68 | 36(16-56) | 42/26 | 31(18-54) | 100 |
| Wadhwani[55] | India | 2020 | RC | 2013-2016 | 20 | 30(18-62) | 16/4 | 35*(nr) | nr |
| Wadhwani[55] | India | 2020 | RC | 2013-2016 | 15 | 30(20-65) | 14/1 | 35*(nr) | nr |
| Brauns[56] | Belgium | 2020 | RS | 2005-2013 | 10 | 46(26-61) | 9/1 | nr | nr |
| Abdou[57] | USA | 2020 | RS | nr | 10 | 37(16-58) | 8/2 | nr | 100 |
| Abula[58] | China | 2020 | RS | 2010-2017 | 14 | 36(18-54) | 8/6 | 30(24-36) | 100 |
| Baruah[59] | India | 2020 | RC | 1997-2010 | 46 | 37(12) | nr | nr | 100 |
| Baruah[59] | Inida | 2020 | RC | 1997-2010 | 40 | 38(12) | nr | nr | 100 |
| Li[60] | China | 2021 | RS | 2014-2019 | 12 | 37(23-54) | 10/2 | nr | 100 |
| Xu[61] | China | 2021 | RS | 2009-2016 | 31 | 33(18-54) | 27/4 | 32(nr) | 100 |
| Xiayimaierdan  [62] | China | 2021 | PC | 2015-2018 | 50 | 41(6) | 24/26 | nr | nr |
| Xiayimaierdan[62] | China | 2021 | PC | 2015-2018 | 50 | 40(4) | 27/23 | nr | nr |
| Huang[63] | China | 2021 | RC | 2015-2018 | 41 | 39(5) | 26/15 | nr(>48) | 100 |
| Huang[64] | China | 2022 | RC | 2013-2018 | 32 | 36(9) | 26/6 | 31(nr) | 100 |
| Rollo[65] | Italy | 2021 | RS | 2006-2018 | 20 | 40(4) | 18/2 | 20 (1-5) | 100 |
| Kliushin[66] | Russia | 2021 | RS | 2005-2014 | 31 | 42*(17-67) | nr | nr | nr |
| Kliushin[66] | Russia | 2021 | RS | 2005-2014 | 18 | 42*(17-67) | nr | nr | nr |
| Hamiti[67] | China | 2022 | RC | 2010-2018 | 18 | 41(11) | 12/6 | 29(nr) | 100 |
| Rohilla[68] | India | 2022 | RCT | 2016-2018 | 13 | 32(18-60) | 12/1 | 32(nr) | 100 |
| Arfee[69] | India | 2022 | PS | nr | 20 | nr(20-50) | nr | nr | nr |
| Arfee[69] | India | 2022 | PS | nr | 20 | nr(20-50) | nr | nr | nr |
| Yushan[70] | China | 2022 | PS | 2017-2019 | 12 | 37(8) | 9/3 | 29(3) | nr |
| Abulaiti[71] | China | 2022 | RC | 2013-2019 | 32 | 38(12) | 23/9 | nr | nr |
| Abulaiti[71] | China | 2022 | RC | 2013-2019 | 21 | 40(13) | 16/5 | nr | nr |
| **Femur** | | | | | | | | | |
| Krishnan[72] | India | 2006 | RS | 1989-2002 | 20 | 38(18-65) | 17/3 | 63(29-110) | 100 |
| Saridis[73] | Greece | 2006 | RS | 1993-2001 | 13 | 35(19-55) | 10/3 | 42(19-72) | 100 |
| Arora[74] | India | 2012 | RS | 2004-2008 | 13 | 29(18-47) |  | 19(15-41) | nr |
| Wan[75] | China | 2013 | RC | 1994-2008 | 13 | 33(5) | 8/5 | 81(6) | 84.6 |
| Yin[33] | China | 2015 | RS | 2004-2013 | 38 | 39(18-62)* | 32/6* | 23(14-46)* | 92.1 |
| Agrawal[76] | India | 2016 | PS | 2010-2012 | 30 | nr(22-62) | 28/2 | nr | 100 |
| Liu[77] | China | 2019 | RS | 1999-2010 | 15 | 33(18-58) | 7/8 | nr | 100 |
| Sen[78] | Turkey | 2019 | RC | 2003-2014 | 17 | 39(26-56) | 12/5 | 66(24-180) | nr |
| Sen[78] | Turkey | 2019 | RC | 2003-2014 | 15 | 42(28-58) | 10/5 | 70(24-240) | nr |
| Bakhsh[79] | Pakistan | 2019 | RS | 2015-2018 | 50 | 34(17-54) | 48/2 | 42 | nr |
| Rohilla[80] | India | 2022 | RS | 2015-2018 | 26 | 32(20-55) | 26/0 | 32 | 100 |

SD: standard deviation; RS: retrospective case series, PS: prospective case series, RC: retrospective control study, PC: prospective control study, RCT: randomized control clinical trial, nr: not reported

* the patients included in the study was part of the original group, while the information marked was extracted from the original group.

**Appendix 3.** Details of Ilizarov method without combinations

| Author | Number | Etiology | Type | Frame | Flap | Defects size (cm) (range/SD) | Mean delay from injury  (mths) (range) | Mean latency  (days) (range) | Mean previous operation  (range/SD) |
| --- | --- | --- | --- | --- | --- | --- | --- | --- | --- |
| **Tibia** |  |  |  |  |  |  |  |  |  |
| Paley[1] | 25 | 7A+18I | 25BT | RF | N | 7.3(1.0-23.0) | 42(5-144) | nr | 3.0(1.0-10.0) |
| Cattaneo[2] | 28 | 28I | 18BT+10ASL | RF | N | nr | 25(4-114) | nr | 3.0(1.0-6.0) |
| Cierny[3] | 21 | 21I | 21BT | RF | sF | 6.5(>4.0) | nr | 7(5-10) | nr |
| Marsh[4] | 10 | 10I | 10BT | RF/UF | 1F | 4.1(nr) | 21(nr) | nr | 5.9(nr) |
| Hosny[5] | 11 | 11I | 3BT+8ASL | RF | 3F | 3.7(1.5-4.9) | 9(6-16) | nr | 2.0(1.0-4.0) |
| Ring[6] | 10 | 10I | 10BT | RF | N | 4.3(1.0-18.0) | 24(3-92) | nr | 2.0(1.0-5.0) |
| Paley[7] | 19 | 11T+8I | 19BT | RF | 4F | 3.9(2.0-15.0) | nr | 7 | 5.0(nr) |
| Maini[8] | 23 | 23I | 23BT | RF/UF | N | 7.7(4.0-12.0) | nr | 7(5-10) | nr |
| Atesalp[9] | 14 | 14I | 14BT | RF | 3F | 4.4(2.5-8.0) | 20(5-60) | nr | nr(3.2-7.0) |
| Bobroff[10] | 12 | 5T+7I | 12BT | RF | N | 9.45(4.0-20.0) | 23(3-100) | nr | 1.4(1.0-3.0) |
| Sen[11] | 24 | 24T | 24ASL | RF | N | 5.0(3.0-8.5) | 8(4-26) | 10 | nr |
| Mekhail[12] | 15 | 9T+6I | 15BT | RF | N | 7.0(2.5-12.0) | 8(1-44) | nr | nr |
| Mahaluxmivala  [13] | 12 | 8T+4I | 6BT+6ASL | RF | 1F | 5.3(3.0-6.0) | 16(6-18) | nr | 1.1(nr) |
| Rozbruch[14] | 25 | 25T | 25BT | RF | N | 6.0(2.0-14.0) | 4(0-24) | nr | nr |
| Baruah[15] | 25 | 25I  25 | 25BT | UF | 7F | 4.36(2.0-9.0) | nr | nr | nr |
| Baruah[15] | 25 | 25I | 25BT | HF | 7F | 2.7(nr) | nr | nr | nr |
| Emara[16] | 16 | 16I | 16BT | UF | N | >4.0 | 13(6-22) | 10 | nr |
| Madhusudhan  [17] | 13 | 13I | 13BT | RF | N | nr(2.0-9.0) | 8(nr) | nr(5-7) | 3.0(2.0-5.0) |
| Pirwani[18] | 16 | 16I | 16BT | RF | N | 4.5(2.0-8.0) | nr | nr | nr |
| Bumbasirević  [19] | 30 | 30I | 30BT | RF | N | 5.7(1.2-6.9) | 9(6-24) | 7 | 1.3(1.0-3.0) |
| Wu[20] | 25 | 25I | 25BT/ASL | RF | N | nr | 12(8-16) | nr | nr |
| Liodakis[21] | 21 | 7T+14I | 21BT | RF | sF | 8.1(2.8) | nr | 5 | 5.7(2.2) |
| Chim[22] | 28 | 5T+18I | 28BT | RF | Y | 6.3(3.0-14.0) | nr |  | 5.0-7.0 |
| Lin[23] | 16 | 16I | 16BT | RF | N | 8.0(4.0-12.0) | nr | 7 | nr |
| Babar[24] | 17 | nr | 17BT | RF | N | 5.8(nr) | nr | 7 | nr |
| Sala[25] | 18 | 5A+13I | 18BT | RF/UF | N | 9.1*(3.5-17.5) | nr | nr | nr |
| Xu[26] | 30 | 30I | 30BT | RF | N | 6.4(3.0-12.0) | 9(6-24) | 7 | 6.0(3.0-14.0) |
| Feng[27] | 21 | 21I | 21BT | RF | N | 6.6(3.0-12.0) | 9(6-24) | 7 | 6.0(3.0-14.0) |
| Krappinger[28] | 15 | 15I | 15BT | RF | 12F | 6.6(3.0-14.7) | 13(1-41) |  | 10.1(2.0-35.0) |
| Selim[29] | 10 | 10T | 10BT | RF | 2F | 9.0(6.0-12.0) | nr | 7 | nr |
| Atef[30] | 28 | 28I | 15BR+13ASL | RF | N | 4.5(nr) | 1(1-2) | 7 | nr |
| Yin[31] | 66 | 66I | 66BT | RF | N | 6.3(3.0-13.0) | 23(4-110) | 7-10 | 2.4(1.0-8.0) |
| Morsy[32] | 12 | 7A+5I | 12BT | RF | N | 4.6(4.0-7.0) | 18(7-26) | 7-10 | nr |
| Yin[33] | 72 | 72I | 72BT | RF | N | 6.15*(3.0-13.0) | 26*(4-110) | 7-10 | 2.61(1.0-8.0)* |
| Ajmera[34] | 30 | 30T | 30BT | UF | 2F | 5.5(4.0-9.0) | nr | 7 | nr |
| Azzam[35] | 30 | 8T+22I | 30BT | RF | N | 7.4(3.0-12.0) | nr | 7 | nr |
| Bernstein[36] | 30 | 14A+16I | 30BT | RF | 13F | 5.7(1.6-12.0) | nr | 7 | nr |
| Fürmetz[37] | 25 | 2A+23I | 25BT | UF/HF | 6F | 6.6(3.0-13.4) | nr | 7 | nr |
| Rohilla[38] | 35 | 35I | 33BT+2ASL | RF | N | 5.8(3.0-9.0) | 7(1-25) | 7 | 1.0 |
| Rohilla[38] | 35 | 35I | 34BT+1ASL | UF | N | 5.8(3.0-10.0) | 8(1-34) | 7 | 1.0 |
| Sadek[39] | 14 | 14I | 14BT | RF | 8F | 4.9(3.0-6.0) | 17(8-34) | 5-7 | 3.3(2.0-6.0) |
| Meleppuram[40] | 42 | 42I | 42BT | RF | N | nr(2.5-5.5) | nr | 7 | nr |
| Tesworth[41] | 21 | 21I | 21BT | RF | 2F | nr(3.0-10.0) | nr | nr | 4.0(nr) |
| Wang[42] | 15 | 15I | 15BT | RF | N | 7.5(3.0-12.0) | nr | 5 | 2.9(1-6) |
| Xu[43] | 18 | 18I | 18BT | RF/UF | Y | 4.5(2.0-6.0) | 2(1-3) | 7 | nr |
| Zhang[44] | 16 | 16I | 16BT | RF | 3F | 10.9(6.0-20.0) | 17(3-45) | 7-10 | 4.3 |
| Wu[45] | 23 | 23T | 23BT | RF | Y | 6.4(4.3-10.0) | nr | 7 | nr |
| Wu[45] | 17 | 17T | 17ASL | RF | Y | 6.7(4.8-11.0) | nr | 7 | nr |
| Catagni[46] | 45 | 26T+19I | 45BT | RF | N | 12.5(9.6-14.4) | nr | 7-10 | nr |
| Catagni[46] | 41 | 16T+25I | 41BT | RF | N | 13.5(10.5-16.0) | nr | 7-10 | nr |
| Rohilla[47] | 15 | 15I | 15BT | UF | N | 4.9(3.0-7.0) | nr | 7 | nr |
| Rohilla[47] | 15 | 15I | 15BT | RF | N | 5.6(3.0-7.0) | nr | 7 | nr |
| Fahad[48] | 51 | 51I | 51BT | RF | 8F | 3.5(2.0-5.0) | nr | 5-7 | 2.0(0.0-14.0) |
| Yikemu[49] | 78 | 78I | 78BT | RF | N | 9.2(6.0-17.0) | nr | 7 | nr |
| Bakhsh[50] | 56 | 56I | 56BT | RF | 7F | 4.3(3.0-9.0) | nr | 7 | nr |
| Kinik[51] | 30 | 30I | 30BT | RF | N | >6.0 | 13(2-36) | 7-10 | 2.9(1.0-9.0) |
| Jilani[52] | 22 | 22I | 8BT+14SAT | UF | sF | 4.7(2.0-9.0) | 22(6-228) | 7 | 2.4(1.0-5.0) |
| Li[53] | 13 | 13T | 13BT | UF | sF | 7.3(5.8-9.0) | nr | 14 | nr |
| Li[53] | 13 | 13T | 13BT | UF | sF | 10.7(7.5-15.0) | nr | 14 | nr |
| Li[54] | 68 | 68T | 21BT+47ASL | RF/UF | 25F | 8.0(4.0-18.0) | 1(0-3) | 7 | nr |
| Wadhwani[55] | 20 | 20I | 20BT | UF | 3F | 4.6(2.0-6.0) | nr | 7-9 | nr |
| Wadhwani[55] | 15 | 15I | 15BT | UF | 2F | 7.6(6.5-10.0) | nr | 7-9 | nr |
| Brauns[56] | 10 | 10I | 10BT | RF | N | 7.0(4.0-12.0) | 14(nr) | 7 | nr |
| Abdou[57] | 10 | 3A+7I | 10BT | nr | Y | 7.1(2.0-12.0) | nr | nr | nr |
| Abula[58] | 14 | 14T | 14BT | RF/UF | Y | 7.0(4.0-12.5) | 4(nr) | nr | 3.4 (2.0-6.0) |
| Baruah[59] | 46 | 46I | 46ASL | RF | sF | nr | nr | 7-10 | nr |
| Baruah[59] | 40 | 40I | 40BT | RF | sF | nr | nr | 7-10 | nr |
| Li[60] | 12 | 12T | 12BT | RF/UF | Y | 8.3(4.0-18.0) | nr | 7 | nr |
| Xu[61] | 31 | 31T | 31BT | RF | N | 11.4(8.0-18.2) | nr | nr | 2.7(2.0-4.0) |
| Xiayimaierdan  [62] | 50 | 21T+29I | 50BT | RF | sF | 4.3(2.0-8.0) | nr | 7 | 2.3(0.9) |
| Xiayimaierdan  [62] | 50 | 24T+26I | 50BT | RF | sF | 4.1(2.0-8.0) | nr | 7 | 2.1(0.9) |
| Huang[63] | 41 | 23T+18I | 41BT | RF | N | 13.1(6.0-22.0) | nr | 7 | nr |
| Huang[64] | 32 | 15T+17I | 32ASL | RF | sF | 6.5(3.0-10.0) | nr | 7 | nr |
| Rollo[65] | 20 | nr | 20BT | RF | Y | 9.2(5.2-15.3) | nr | 7 | nr |
| Kliushin[66] | 31 | 31I | 31BT | RF | N | 2.3*(2.0-10.0) | nr | nr | nr |
| Kliushin[66] | 18 | 18I | 18BT | RF | N | nr(1.0-2.0) | nr | nr | nr |
| Hamiti[67] | 18 | 18I | 18BT | nr | N | 6.9(0.7) | nr | nr | 2.0(1.0-3.0) |
| Rohilla[68] | 13 | 13I | 13BT | RF/UF | N | 3.9(3.0-6.0) | nr | 7 | 1(nr) |
| Arfee[69] | 20 | 20I | nr | RF | N | nr | nr | nr | nr |
| Arfee[69] | 20 | 20I | nr | UF | N | nr | nr | nr | nr |
| Yushan[70] | 12 | 4T+8I | 12BT | RF/UF | sF | 7.1(6.3-8.2) | nr | 7-10 | nr |
| Abulaiti[71] | 32 | 32I | 32BT | UF | sF | 7.8(1.8) | nr | 7 | 2.5(0.9) |
| Abulaiti[71] | 21 | 21I | 21BT | UF | sF | 9.4(1.5) | nr | 7 | 2.6(1.1) |
| **Summary** | **2169** | **-** | **-** | **-** | **-** | **6.65(2.7-13.5)** | **-** | **-** | **2.1(1-6)** |
| **Femur** |  |  |  |  |  |  |  |  |  |
| Krishnan[72] | 20 | 20I | 11BT+9ASL | RF | N | 6.0(2.0-10.5) | 10(3-24) | 7(7-10) | 4.4(1.0-9.0) |
| Saridis[73] | 13 | 13T | 13BT | RF | 1F | 8.3(3.0-18.0) | 12(3-33) | 3-5 | 3.0(1.0-5.0) |
| Arora[74] | 13 | 13T | 13BT | UF | N | 7.9(5.5-1.7) | 8(6-18) | 1-7 | 2.9(nr) |
| Wan[75] | 13 | 4T+9I | 13BT | UF | N | 8.9(2.3) | nr | 7 | 2.6(1.3) |
| Yin[33] | 38 | 38I | BT | RF | N | 6.2*(3.0-13.0) | 26(4-110)* | 7-10 | 2.6(1.0-8.0)* |
| Agrawal[76] | 30 | 30(A+I) | 26BT+4ASL | UF | sF | 5.8(2.0-16.0) | nr | 7 | 2.2(1.0-4.0) |
| Liu[77] | 15 | 15I | 15BT | UF | N | 8.7(4.0-16.0) | 26(14-38) | 3-5 | 4.8(1.0-17.0) |
| Sen[78] | 17 | 17I | 17ASL | UF | N | 5.5(3.0-10.0) | nr | 7 | 3.9(3.0-6.0) |
| Sen[78] | 15 | 15I | 15BT | RF | N | 5.9(3.0-10.0) | nr | 7 | 2.4(1.0-12.0) |
| Bakhsh[79] | 50 | 50(A+I) | 50BT | RF | N | 3.6(1.0-7.0) | 11(10-20) | 7 | 2.5(1.0-11.0) |
| Rohilla[80] | 26 | 26I | 26BT | UF | N | 4.3(2.0-7.0) | 30(24-65) | 7 | 2.8(1.0-4.0) |
| **Summary** | **250** | **-** | **-** | **-** | **-** | **5.8(3.6-8.9)** | **-** | **-** | **2.9(2.2-4.8)** |

SD: standard deviation; A: aseptic lesion, T: trauma lesion, I: infected lesion, BT: bone transport, ASL: acute shorting and lengthening, RF: ring frame, UF: unilateral frame, F: flaps, sF: some of patients applied flaps, nr: not reported

* the patients included in the study was part of the original group, while the information marked was extracted from the original group.

**Appendix 4.** Interested outcomes of included studies (ilizarov method without combinations)

| Author | Number | Union rate (%) | Bone results  (excellent/good  /fair/poor) | Functional results(excellent/good/fair/poor) | EFT  (mths)  (range/SD) | EFI  (mths/cm)  (range/SD) | Bone graft  (n, %) | Complications  (per patient) |
| --- | --- | --- | --- | --- | --- | --- | --- | --- |
| **Tibia** |  |  |  |  |  |  |  |  |
| Paley[1] | 25 | 100.0 | 18/5/2/0 | 16/7/1/0 | nr | nr | 0 | 0.7(18/25) |
| Cattaneo[2] | 28 | 89.3 | nr | nr | nr | nr | 0 | nr |
| Cierny[3] | 21 | 100.0 | nr | nr | nr | nr | 14(67) | 0.3(7/21) |
| Marsh[4] | 10 | 80.0 | nr | nr | 8.7(nr) | nr | 1(10) | 2.1(21/10) |
| Hosny[5] | 11 | 100.0 | nr | 5/3/2/1 | 8.5(5.5-16) | nr | 0 | 1.2(13/11) |
| Ring[6] | 10 | 90.0 | nr | nr | nr | nr | 3(30) | 2.3(23/10) |
| Paley[7] | 19 | 100.0 | nr | 12/6/0/1 | 16(6-22) | 1.7(0.7-4.3) | 7(37) | 1.2(22/19) |
| Maini[8] | 23 | 100.0 | 15/3/0/5 | nr | nr | nr | 3(10) | 1.7(39/23) |
| Atesalp[9] | 14 | 68.4 | nr | nr | nr | nr | 0 | 1.2(17/14) |
| Bobroff[10] | 12 | 100.0 | 6/3/0/3 | 6/2/2/2 | 16.7(2.0-25.5) | 1.5(1.0-2.3) | 9(75) | 1.1(13/12) |
| Sen[11] | 24 | 100.0 | 21/3/0/0 | 19/4/1/0 | 7.1(3-10) | 1.4(nr) | 0 | 2.2(52/24) |
| Mekhail[12] | 15 | 93.3 | 1/9/3/1 | nr | 12.9(4.6-23) | nr | 18(75) | 2.1(32/15) |
| Mahaluxmivala  [13] | 12 | 100.0 | nr | nr | 7.5(8.0-24.0) | nr | 6(33) | nr |
| Rozbruch[14] | 25 | 96.0 | nr | nr | nr | nr | 12(48) | 0.8(21/25) |
| Baruah[15] | 25 | 100.0 | nr | nr | 6.5(3.3-14.8) | nr | 0 | 0.8(20/25) |
| Baruah[15] | 25 | 100.0 | nr | nr | 5.7(3.7-9.5) | nr | 0 | 0.4(11/25) |
| Emara[16] | 16 | 100.0 | 15/1/0/0 | 12/1/3/0 | 8.5(6.4-11.0) | 1.5(1.4-1.6) | 16(100)* | 0.4(6/16) |
| Madhusudhan  [17] | 13 | 100.0 | 5/8/5/4 | 1/4/6/10 | 9.3(6.5-13.0) | nr | 0 | 2.7(60/22) |
| Pirwani[18] | 16 | 100.0 | nr | nr | 16.0(nr) | 3.0(nr) | nr | 2.0(32/16) |
| Bumbasirević  [19] | 30 | 96.7 | 19/10/1/0 | 13/14/2/1 | 9.7(5.8-15.0) | 1.5(1.4-1.7) | 1(3) | 1.4(43/30) |
| Wu[20] | 25 | 100.0 | nr | nr | nr | nr | 25(100)* | nr |
| Liodakis[21] | 21 | 90.5 | nr | nr | 15.9(6.1) | nr | nr | nr |
| Chim[22] | 28 | 92.9 | nr | nr | nr | nr | 28(100)* | 0.6(17/28) |
| Lin[23] | 16 | 93.8 | nr | nr | 4.5(nr) | nr | 16(100)* | 1.0(16/16) |
| Babar[24] | 17 | 94.1 | 13/2/2/0 | 10/4/2/1 | 6.0(nr) | nr | nr | 1.0(17/17) |
| Sala[25] | 18 | 100.0 | 11/7/0/0 | 8/8/2/0 | 15.5(nr) | 1.8(1.1-3.4) | 10(83) | 0.3(6/18) |
| Xu[26] | 30 | 100.0 | 28/2/0/0 | nr | 10.0(8.0-14.0) | 1.4(nr) | nr | 0.3(8/30) |
| Feng[27] | 21 | 100.0 | 19/2/0/0 | nr | 9.8(nr) | 1.48(nr) | nr | 0.4(8/21) |
| Krappinger[28] | 15 | 100.0 | 7/6/2/0 | 6/7/2/0 | 13.2(7.0-25.0) | nr | 15(100)* | 1.4(20/14) |
| Selim[29] | 10 | 80.0 | 7/3/0/0 | 7/3/0/0 | 2.5(0.28) | nr | 2(20) | 0.7(7/10) |
| Atef[30] | 28 | 96.4 | 16/9/2/1 | nr | 10.0(7.0-14.0) | 1.5(1.0-2.3) | 28(100)* | 0.9(25/28) |
| Yin[31] | 66 | 100.0 | 44/15/5/2 | 24/26/10/0 | 9.4(nr) | 1.4(nr) | 6(9) | 1.1(73/66) |
| Morsy[32] | 12 | 100.0 | 8/3/0/1 | 7/4/1/0 | 6.8(nr) | 1.5(nr) | nr | 1.6(19/12) |
| Yin[33] | 72 | 100.0 | 46/17/7/2 | 25/27/13/0 | nr | 1.5(1.3-1.7) | 7(58) | nr |
| Ajmera[34] | 30 | 92.0 | 19/3/1/2 | 21/2/2/0 | 11(6.0-12.8) | nr | 2(7) | 2.1(52/25) |
| Azzam[35] | 30 | 100.0 | 22/6/1/1 | 13/9/7/1 | 7.5(4.5-11.5) | nr | 30(100)* | 2.2(51/23) |
| Bernstein[36] | 30 | 83.3 | nr | nr | 11.0(4.5-19.6) | 2.5(nr) | 1(3) | 0.8(17/22) |
| Fürmetz[37] | 25 | 92.0 | nr | nr | 8.5(3.5-21.2) | 1.3(nr) | 15(60) | 1.9(48/25) |
| Rohilla[38] | 35 | 97.1 | 21/12/2/0 | 16/17/2/0 | 11.1(nr) | 2.1(nr) | 0 | 1.7(60/35) |
| Rohilla[38] | 35 | 94.3 | 14/15/6/0 | 22/10/3/0 | 10.5(nr) | 2.1(nr) | 0 | 2.0(70/35) |
| Sadek[39] | 14 | 100.0 | 11/3/0/0 | 8/3/3/0 | 7.8(3.0-15.0) | 1.6(0.6-2.0) | 4(29) | 0.6(8/14) |
| Meleppuram[40] | 42 | 100.0 | 60/15/25/0 | 55/30/5/10 | nr (8.0-10.0) | nr | 13(31) | 1.4(58/42)（38problems 2in 42patients；20 obstacles in 18 patients） |
| Tesworth[41] | 21 | 100.0 | 15/5/1/0 | 19/2/0/0 | 12.5(6.0-23.0) | 1.8(nr) | 14(67) | 2.7(57/21) |
| Wang[42] | 15 | 100.0 | nr | nr | 12.0(10.0-20.7) | 1.4(1.1-2.1) | 6(40) | 1.9(28/15) |
| Xu[43] | 18 | 100.0 | nr | nr | 11.4(7.0-20.0) | nr | 10(56) | nr |
| Zhang[44] | 16 | 100.0 | 10/0/0/6 | 12/4/0/0 | 12.0(nr) | 1.1(nr) | 3(19) | 1.2(19/16) |
| Wu[45] | 23 | 100.0 | 23/5/0/0 | 11/10/7/0 | nr | nr | 12(52) | 0.7(15/23) |
| Wu[45] | 17 | 100.0 | 18/4/0/0 | 7/10/5/0 | nr | nr | 2(12) | 0.7(12/17) |
| Catagni[46] | 45 | 100.0 | nr | nr | 11.5*(9.0-12.5) | nr | 16(36) | 2.3(104/45) |
| Catagni[46] | 41 | 100.0 | nr | nr | 8.7*(7.6-10.6) | nr | 32(78) | 2.1(87/41) |
| Rohilla[47] | 15 | 80.0 | 7/7/0/1 | 11/3/0/1 | 7.8(5.0-11.0) | 1.6(nr) | 0 | 1.1(16/15) |
| Rohilla[47] | 15 | 73.3 | 11/1/2/1 | 7/6/2/0 | 8.6(5.0-18.0) | 1.7(nr) | 0 | 0.9(13/15) |
| Fahad[48] | 51 | 96.1 | 22/19/7/3 | 24/21/5/1 | 10.0(nr) | 2.0(1.5-4.0) | nr | 0.7(35/51) |
| Yikemu[49] | 78 | 100.0 | 59/14/5/0 | nr | nr | nr | nr | nr |
| Bakhsh[50] | 56 | 98.2 | 37/10/6/3 | 37/9/7/3 | 9.3(nr) | 1.4(1.2-1.6) | nr | 0.9(50/56) |
| Kinik[51] | 30 | 100.0 | 22/6/0/0 | 19/7/2/0 | 13.7(nr) | 1.5(nr) | 1(3) | 2.0(59/30) |
| Jilani[52] | 22 | 90.9 | 12/5/3/2 | 11/5/4/2 | nr | nr | 2(9) | 1.8(40/22) |
| Li[53] | 13 | 100.0 | 9/0/0/4 | 11/0/0/2 | 18.1(15.0-20.0) | 2.5(2.2-2.9) | nr | nr |
| Li[53] | 13 | 100.0 | 10/3/0/0 | 12/1/0/0 | 12.7(9.5-16.0) | 1.2(1.0-1.7) | nr | nr |
| Li[54] | 68 | 79.4 | nr | 34/18/10/6 | 12.5(3.4) | 1.6(0.44) | nr | 1.0(66/68) |
| Wadhwani[55] | 20 | 90.0 | 14/5/1/0 | 15/4/1/0 | 9.2(nr) | 2.1(nr) | nr | 0.9(17/20) |
| Wadhwani[55] | 15 | 73.3 | 4/6/5/0 | 5/8/2/0 | 12.2(nr) | 2.1(nr) | nr | 1.5(23/15) |
| Brauns[56] | 10 | 100.0 | 7/2/1/0 | 5/4/1/0 | 15.0(11.0-22.0) | nr | 4(40) | nr |
| Abdou[57] | 10 | 100.0 | nr | nr | 8.2(1.6-22.9) | nr | 0 | 2.0(12/6) |
| Abula[58] | 14 | 100.0 | 8/6/0/0 | 8/6/0/0 | 7.0(5.6-7.8) | 1.1(0.6-1.6) | 4(29) | nr |
| Baruah[59] | 46 | 100.0 | 10/24/8/4 | 8/26/10/2 | nr | nr | nr | 0.2(9/46) |
| Baruah[59] | 40 | 92.5 | 6/18/10/6 | 5/18/12/5 | nr | nr | nr | 1.1(42/40) |
| Li[60] | 12 | 80.0 | nr | 24/10/4/2 | 12.8(10.0-24.0) | 1.6(nr) | 8(67) | 1.0(41/40) |
| Xu[61] | 31 | 100.0 | 6/14/8/3 | 8/15/5/3 | 22.7(14.0-37.0) | 1.9(1.2-2.5) | 4(13) | 1.6(50/31) |
| Xiayimaierdan  [62] | 50 | 100.0 | 15/9/15/11 | 14/10/16/10 | 16.6(3.9) | nr | 50(100)* | 0.2(10/50) |
| Xiayimaierdan  [62] | 50 | 100.0 | 20/14/12/3 | 17/18/13/2 | 9.4(2.2) | nr | 50(100)* | 0.1(3/50) |
| Huang[63] | 41 | 100.0 | 23/11/2/0 | 14/9/13/0 | 10.1(2.0) | 1.4(0.3) | 0 | 0.8(34/41) |
| Huang[64] | 32 | 100.0 | 22/7/3/0 | 15/7/10/0 | 9.2(1.8) | 1.5(0.2) | 12(38) | 1.5(49/32) |
| Rollo[65] | 20 | 100.0 | 8/6/6/0 | nr | 19.2(9.0-32.0) | 1.0(0.4-1.8) | nr | 1.0(21/20) |
| Kliushin[66] | 31 | 80.6 | nr | nr | 4.7(2.4) | nr | nr | nr |
| Kliushin[66] | 18 | 72.2 | nr | nr | 2.5(1.8) | nr | nr | nr |
| Hamiti[67] | 18 | 100.0 | 7/7/3/1 | 5/8/3/2 | 9.8(0.4) | 1.2(0.1) | nr | 3.2(57/18) |
| Rohilla[68] | 13 | 92.3 | 9/3/1/0 | 8/4/1/0 | 9.42(6.0-12.0) | 2.5(0.6) | nr | 1.5(20/13) |
| Arfee[69] | 20 | 100.0 | 15/4/1/0 | 16/3/1/0 | nr | nr | 0 | nr |
| Arfee[69] | 20 | 100.0 | 13/4/3/0 | 12/4/4/0 | nr | nr | 0 | nr |
| Yushan[70] | 12 | 100.0 | 7/4/0/1 | 5/6/0/1 | 5.2(0.8) | 0.7(0.2) | 2(17) | 1.7(20/12) |
| Abulaiti[71] | 32 | 100.0 | 13/15/3/1 | 11/8/2/0 | 15.8(3.44) | 2.0(0.1) | 1(3) | 2.9(94/32) |
| Abulaiti[71] | 21 | 100.0 | 10/16/6/0 | 8/10/3/0 | 10.9(1.9) | 1.2(0.1) | 0 | 1.7(36/21) |
| **Summary** | **2169** | **95.8** | **-** | **-** | **11.0(2.5-34.7)** | **1.6(0.7-3.0)** | **269(21)** | **1.2(2090/1680)** |
| **Femur** |  |  |  |  |  |  |  |  |
| Krishnan[72] | 20 | 95.0 | 13/4/1/1 | 3/9/3/4 | 7.8(2.6-14.9) | nr | 1(5) | 3.6(71/20) |
| Saridis[73] | 13 | 92.3 | 8/4/1/0 | 3/4/4/2 | 10.3(4.5-20.2) | nr | 6(46) | 1.8(23/13) |
| Arora[74] | 13 | 100.0 | 12/3/0/0 | 5/8/2/0 | 7.3(nr) | 0.9(nr) | 3(23) | 2.7(35/13) |
| Wan[75] | 13 | 100.0 | 6/2/5/0 | 7/4/2/0 | 14.8(6.9) | 4.4(0.1) | 3(23) | 2.0(26/13) |
| Yin[33] | 38 | 100.0 | 22/11/5/0 | 12/15/8/0 | nr | 1.5(1.2-1.7) | 7(18) | nr |
| Agrawal[76] | 30 | 100.0 | 17/9/3/1 | 9/14/5/2 | nr | nr | 5(17) | 2.0(59/30) |
| Liu[77] | 15 | 100.0 | 13/2/0/0 | 6/6/3/0 | nr | 1.4(1.2-1.6) | nr | 2.3(35/15) |
| Sen[78] | 17 | 100.0 | 11/4/3/0 | 10/5/2/0 | nr | nr | 1(6) | 1.6(27/17) |
| Sen[78] | 15 | 100.0 | 9/5/1/0 | 9/5/1/0 | nr | nr | 4(27) | 3.1(47/15) |
| Bakhsh[79] | 50 | 96.0 | 17/30/1/2 | 15/24/8/3 | nr | nr | nr | 1.9(95/50) |
| Rohilla[80] | 26 | 84.6 | 15/9/0/2 | 11/13/1/1 | 10.9(6.0-18.0) | 2.8(2.0-6.0) | 3(12) | 1.1(28/26) |
| **Summary** | **250** | **97.0** | **-** | **-** | **11.4(7.3-14.8)** | **2.1(0.9-4.4)** | **33(18)** | **2.0(460/232)** |

SD: standard deviation; EFT: external frame time, EFI: external frame index, nr: not reported

Bone result and functional result were evaluated by ASAMI (Association for the Study of the Method of Ilizarov).

* active management of bone grafting was applied, and relevant studies were excluded when calculated the mean rate of bone grafting used.

**Appendix 5.** Descriptive characteristics of included studies (Ilizarov technique combined with antibiotic spacer)

| Author | Country | Publishedyear | Design | Treatment period | Number | Age  (range) | Male/  female | Mean follow-up time (mths) (range/SD) | Follow-up  rate (%) |
| --- | --- | --- | --- | --- | --- | --- | --- | --- | --- |

| **Tibia** | | | | | | | | | |
| --- | --- | --- | --- | --- | --- | --- | --- | --- | --- |
| Hutson Jr[81] | USA | 2010 | RS | 1992-2008 | 18 | 32(23-50) | 13/5 | 66(8-138) | 100 |
| Spiegl[82] | Germany | 2013 | PS | 2006-2009 | 25 | 46(20-60) | 22/3 | 29(nr) | 100 |
| Peng[83] | China | 2015 | RS | 2008-2011 | 58 | 29(18-51) | 38/20 | 31(24-63) | 100 |
| van Niekerk[84] | South Africa | 2017 | RC | 2009-2013 | 12 | 33(16-60) | 6/6 | nr | 100 |
| van Niekerk[84] | South Africa | 2017 | RC | 2009-2013 | 12 | 32(16-60) | 10/2 | nr | 100 |
| Hamiti[85] | China | 2021 | RS | 2010-2016 | 18 | 40(22-62) | 12/6 | 29(nr) | 100 |
| Huang[63] | China | 2021 | RC | 2015-2018 | 44 | 39(5) | 30/14 | nr | 100 |
| Liu[86] | China | 2022 | RS | 2011-2017 | 21 | 47(7) | 13/8 | 27(3) | 100 |
| Khaled[87] | Egypt | 2022 | PS | 2012-2019 | 32 | 24(19-52) | 27/5 | 28(16-36) | 100 |
| Hamiti[67] | China | 2022 | RC | 2010-2018 | 18 | 41(11) | 14/4 | 27(25-32)* | 100 |
| Xu[88] | China | 2022 | RC | 2018-2021 | 21 | 49(25-67) | 15/6 | 17(9-31) | 100 |
| **Femur** | | | | | | | | |  |
| Song[89] | China | 2003 | RS | 1996-1998 | 20 | 36(17-65) | 19/1 | 38(30-60) | 100 |
| Sen[90] | China | 2020 | RS | 2003-2018 | 23 | 37(26-56) | 17/6 | 51(18-192) | 100 |
| Peng[91] | China | 2022 | RS | 2008-219 | 76 | 39(23-60) | 58/18 | 31(23-41) | 100 |
| Liu[86] | China | 2022 | RS | 2011-2017 | 18 | 46(6) | 13/5 | 28(4) | 100 |

SD: standard deviation, RS: retrospective case series, PS: prospective case series, RC: retrospective control study, PC: prospective control study, RCT: randomized control clinical trial

* median and interquartile range

**Appendix 6.** Details of Ilizarov technique combined with antibiotic spacer

| Author | Number | Etiology | Ingredients  of spacer | Frame | Defects size  (cm) | Mean delay from injury  (mths) (range/SD) | Mean spacer time (w) (range/SD) | Mean latency  (d, range) | Mean previous operation  (range) |
| --- | --- | --- | --- | --- | --- | --- | --- | --- | --- |
| **Tibia** |  |  |  |  |  |  |  |  |  |
| Hutson Jr  [81] | 18 | nr | Twenty grams of cement is dry mixed with 2.4 g tobramycin powder and 1.0 g of vancomycin | nr | 9.9  (5.0-17.0) | nr | 15.5  (9.0-44.0) | nr | nr |
| Spiegl  [82] | 25 | 25I | Antibiotic-impregnated cement (impregnated with gentamicin for the most part) | UF | 5.3  (3-13) | 10(2-24) | nr | 7 | nr |
| Peng  [83] | 58 | 58I | antibiotic-impregnated cement mixed with tobramycin and vancomycin-ten grams of cement mixed with 1.2 g tobramycin and 0.5 g vancomycin | RF | 9.2  (6.0-15.0) | 31(6-50) | nr | 10 | 6.3  (3.0-10.0) |
| van Niekerk  [84] | 12 | 12I | Sensitive antibiotic-impregnated PMMA spacer | RF | 5.0(2.6) | nr | 9.9(3.6) | 7-10 | nr |
| van Niekerk  [84] | 12 | 12T | Sensitive antibiotic-impregnated PMMA spacer | RF | 8.3(3.6) | nr | 7.7(2.2) | 7-10 | nr |
| Hamiti  [85] | 18 | 18I | PMMA spacer mixed with 10% vancomycin | nr | 7.1(0.8) | nr | nr | nr | 3.3  (1.0-5.0) |
| Huang  [63] | 44 | 25T+19I | calcium sulfate 7.5g + vancomycin 0.5 g + gentamicin injection 3 ml | RF | 12.5(3.6) | nr | nr | nr | nr |
| Liu  [86] | 21 | 21I | Antibiotic-impregnated cement spacer (5 g vancomycin per 40 g gentamicin-loaded bone cement | UF | nr | nr | nr | 7 | nr |
| Khaled  [87] | 32 | 32I | cement spacer (mixing 2 gm vancomycin with 40 gm gentamycin-impregnated cement powder) | RF | 6.0  (4.0-14.5) | nr | nr  (6-8) | 7 | 3.0  (2.0-4.0) |
| Hamiti  [67] | 18 | 18I | PMMA spacer mixed with 10% vancomycin | nr | 7.0(0.8) | nr(4-18) | nr | nr | 2.0  (1.8-3.0) |
| Xu  [88] | 21 | 21I | Antibiotic calcium sulphate spacer with sensitive antibiotics | RF | 8.4  (6.6-10.4) | nr | nr | nr | nr |
| **Summary** | **322** | **-** | **-** | **-** | **4.8**  **(4.5-9.9)** | **-** | **-** | **-** | **4.1**  **(1.9-6.3)** |
| **Femur** |  |  |  |  |  |  |  |  |  |
| Song  [89] | 20 | 20I | PMMA mixed with antibiotics | RF/HF | 10.0  (6.0-22.0) | nr | nr | nr | 4.8  (1.0-17.0) |
| Sen  [90] | 76 | 76I | Antibiotic-impregnated cement spacer (5 g vancomycin per 40 g gentamicin-loaded bone cement) | UF | 4.6  (3.7-5.6) | 30(16-41) | nr | 6 | nr |
| Peng  [91] | 18 | 18I | Antibiotic-impregnated cement spacer (5 g vancomycin per 40 g gentamicin-loaded bone cement) | UF | nr | nr | nr | 7 | nr |
| Liu  [86] | 23 | 25I | Antibiotic impregnated beads or rods (2 g of powdered vancomycin per 40 g of PMMA) | UF | 2.7(nr) | nr | nr | 7 | nr |
| **Summary** | **137** | **-** | **-** | **-** | **5.1**  **(2.7-10.0)** | **-** | **-** | **-** | **4.8(nr)** |

SD: standard deviation; T: trauma lesion, I: infected lesion, RF: ring frame, UF: unilateral frame, nr: not reported, PMMA: polymethyl methacrylate

* the patients included was part of the original group, while the information marked was extracted from the original group.

**Appendix 7.** Interested outcomes of included studies (Ilizarov technique combined with antibiotic spacer)

| Author | Number | Union  rate (%) | Bone results  (excellent/good/fair/poor) | Functional results (excellent/good  /fair/poor) | EFT  (mths)  (range/SD) | EFI  (mths/cm)  (range/SD) | Bone graft  (n, %) | Complications  (per patient) |
| --- | --- | --- | --- | --- | --- | --- | --- | --- |
| **Tibia** |  |  |  |  |  |  |  |  |

| Hutson Jr[81] | 18 | 100.0 | nr | nr | nr | nr | 14(78) | 0.8(14/18) |
| --- | --- | --- | --- | --- | --- | --- | --- | --- |
| Spiegl[82] | 25 | 96.0 | nr | nr | nr | nr | nr | 1.4(35/25) |
| Peng[83] | 58 | 100.0 | 30/23/5/0 | 28/18/12/0 | 10.6(8.0-31.0) | 0.9(0.6-1.4) | 58(100)* | 0.6(37/58) |
| van Niekerk[84] | 12 | 91.7 | nr | nr | 8.5(4.8) | 1.9(0.5) | 12(100)* | nr |
| van Niekerk[84] | 12 | 91.7 | nr | nr | 9.8(3.3) | 1.2(0.3) | 12(100)* | nr |
| Hamiti[85] | 18 | 100.0 | 6/8/3/1 | 4/10/2/2 | 1.2(1.0-1.8) | 1.2(1.0-1.5) | 0 | 2.9(52/18) |
| Huang[63] | 44 | 100.0 | nr | nr | 6.2(1.5) | 0.6(0.1) | 4(9) | 0.3(15/44) |
| Liu[86] | 21 | 100.0 | 2/13/4/2 | 4/13/3/1 | 11.5(0.1) | 2.2(0.1) | 5(24) | 0.8(16/21) |
| Khaled[87] | 32 | 93.8 | nr | nr | nr | 1.5(1.2-2.0) | 1(3) | 1.2(37/32) |
| Hamiti[67] | 18 | 100.0 | 9/6/2/1 | 4/9/3/2 | 9.6(0.5) | 1.2(0.1) | 0 | 2.6(47/18) |
| Xu[88] | 21 | 100 | 11/6/3/1 | 9/6/5/1 | 6.1(4-11) | 0.7(0.6-1.1) | 0 | 0.4(8/21) |
| **Summary** | **297** | **98.3** | **-** | **-** | **8.2(1.2-11.5)** | **1.3(0.6-2.2)** | **46(24)** | **1.0(277/269)** |
| **Femur** | | | | | | | | |
| Song[89] | 20 | 100.0 | 13/1/1/5 | 0/9/8/3 | 9.7(2.0-18.0) | 1.4(nr) | 20(100)* | 0.7(14/20) |
| Sen[90] | 76 | 100.0 | 16/42/15/3 | 20/44/9/3 | 11.8(6.0-21.0) | 1.7(1.4-2.4) | 18(100)* | 1.0(72/72) |
| Peng[91] | 18 | 100.0 | 3/11/3/1 | 3/14/1/0 | 11.5(0.28) | 1.9(0.1) | 1(6) | 0.9(17/18) |
| Liu[86] | 23 | 100.0 | 15/6/2/0 | 14/7/2/0 | 3.8(3.0-5.0) | 1.0(0.7-1.7) | 1(4) | 1.0(23/23) |
| **Summary** | **137** | **100.0** | **-** | **-** | **9.0(3.8-11.8)** | **1.5(1.0-1.9)** | **2(1)** | **1.0(112/113)** |

SD: standard deviation; EFT: external frame time, EFI: external frame index, nr: not reported

Bone result and functional result were evaluated by ASAMI (Association for the Study of the Method of Ilizarov).

* active management of bone grafting was applied, and relevant studies were excluded when calculated the mean rate of bone grafting used.

**Appendix 8.** Descriptive characteristics of included studies (Ilizarov technique combined with internal fixation)

| Author | Country | Published year | Design | Treatment  period | Number | Mean age  (range/SD) | Male/  female | Mean follow-up time (mths) (range/SD) | Follow-up  rate(%) |
| --- | --- | --- | --- | --- | --- | --- | --- | --- | --- |
| **Tibia+** **intramedullary nailing** | | | | | | | | |  |
| Oh[92] | Korea | 2008 | RS | 1996-2004 | 11 | 44(18-59) | 11/0 | nr(25-72) | 100 |
| Liodakis  [93] | Germany | 2010 | RC | 2001-2008 | 14 | 45(14) | 11/3 | 61(25) | 100 |
| Liodakis  [21] | Germany | 2011 | RC | 1992-2007 | 18 | 47(14) | 11/7 | 8(6) | 100 |
| Eralp[94] | Turkey | 2012 | RC | 1998-2000 | 17 | 39(25-69) | 14/3 | 29(9-80) * | 88.2 |
| Bas[95] | Turkey | 2020 | RS | 2000-2018 | 20 | 32(16-49) | 15/5 | 24(12-56)* | 100 |
| Xu[88] | China | 2022 | RC | 2018-2021 | 12 | 47(32-67) | 7/5 | 15(8-21) | 100 |
| **Tibia+ locking plate** | | | | | | | | |  |
| Oh[96] | Korea | 2013 | RS | 2007-2011 | 10 | 40(16-64) | 9/1 | 29(18-62) | 100 |
| Gupta[97] | India | 2018 | RS | 2012-2015 | 14 | 38.1(12.7) | 13/1 | 33(24-50) | 100 |
| Lu[98] | China | 2020 | RS | 2013-1017 | 12 | 45(20-65) | 10/2 | 26(12-48) | 100 |
| **Femur+ intramedullary nailing** | | | | | | | | |  |
| Li[99] | China | 2009 | RS | 1998-2004 | 17 | 24(17-32) | 11/6 | 70(24-96) | 100 |
| Wan[75] | China | 2013 | RC | 1994-2008 | 15 | 33(5) | 9/6 | 78(9) | 100 |
| Bas[95] | Turkey | 2020 | RS | 2000-2018 | 19 | 36(17-64) | 11/8 | 28(12-64) | 100 |
| Borzunov  [100] | Russia | 2022 | RC | 2012-2016 | 14 | 33(2) | 11/3 | 13(6-31) | 100 |

SD: standard deviation; RS: retrospective case series, PS: prospective case series, RC: retrospective control study, PC: prospective control study, RCT: randomized control clinical trial, nr: not reported

* the patients included was part of the original group, while the information marked was extracted from the original group.

**Appendix 9.** Details of Ilizarov technique combined with internal fixation

| Author | Number | Aetiology | Applied  before  distraction? | Frame | Defects size  (cm) | Mean delay from injury (mths) (range/SD) | Mean latency  (day) (range) | Mean previous operation  (range/SD) |
| --- | --- | --- | --- | --- | --- | --- | --- | --- |
| **Tibia+ intramedullary nailing** | | | | | | | | |
| Oh[92] | 11 | 2T+9I | Y | RF/UF | 5.8(3.5-12.0) | nr | 10 | nr |
| Liodakis  [93] | 14 | 14T | Y | nr | 8.0(3.0) | nr | 5 | 4.5(2.9) |
| Liodakis  [21] | 18 | 10I | Y | UF | 8.5(3.4) | nr | 5 | 4.3(2.9) |
| Eralp[94] | 17 | 17I | Y | RF/UF | 8.9(2.6-16.0) | nr | nr | 3.3(1.0-13.0) |
| Bas[95] | 20 | - | Y | RF | 8.4(2.5-16.5) | nr | 7 | nr |
| Xu[88] | 12 | 12T | Y | RF | 8.1(6.0-10.9) | nr | 10 | nr |
| **Summary** | **92** | **-** | **-** | **-** | **8.1(5.8-8.9)** | **-** | **-** | **4.4(4.3-4.5)** |
| **Tibia+ locking plate** | | | | | | | | |
| Oh[96] | 10 | 10I | Y | RF/UF | 5.9(3.8-9.3) | 9(3-17) | 10 | nr |
| Gupta[97] | 14 | 14I | Y | UF | 6.4(1.3) | nr | 10 | nr |
| Lu[98] | 12 | - | Y | RF | 6.7(4.0-9.2) | 5(1-14) | 10 | 2.5(nr) |
| **Summary** | **36** | **-** | **-** | **-** | **6.4(5.9-6.7)** | **-** | **-** | **2.5(nr)** |
| **Femur+ intramedullary nailing** | | | | | | | | |
| Li[99] | 17 | 17I | Y | UF | 11.3(8.0-18.4) | 6(4-9) | 7 | 3.0(1.0-7.0) |
| Wan[75] | 15 | 4T+11I | Y | UF | 8.5(3.1) | 24(6) | 7 | 2.9(1.5) |
| Bas[95] | 19 | - | Y | RF | 5.7(1.0-14.0) | nr | 7 | 2.8(1.0-6.0) |
| Borzunov  [100] | 14 | 14A | Y | nr | 4.5(3.0-8.0) | nr | 5-7 | 2.4(1.0-4.0) |
| **Summary** | **65** | **-** | **-** | **-** | **7.6(4.5-11.3)** | **-** | **-** | **2.8(2.4-3.0)** |

SD: standard deviation; T: trauma lesion, I: infected lesion, Y: Yes, RF: ring frame, UF: unilateral frame, nr: not reported

**Appendix 10.** Outcomes of included studies (Ilizarov technique combined with internal fixation)

| Author | Number | Union  rate (%) | Bone result (excellent/good/fair/poor) | Functional results  (excellent/good/  fair/poor) | EFT  (mths)  (range/SD) | EFI  (mths/cm)  (range/SD) | Bone graft (%) | Complications  (per patient) |
| --- | --- | --- | --- | --- | --- | --- | --- | --- |
| **Tibia+ combined** **intramedullary nailing with Ilizarov** | | | | | | | | |
| Oh[92] | 11 | 100.0 | nr | 5/6/1/0 | 4.7(2.6-7.7) | 0.9(0.5-1.5) | 1(9) | 1.2(14/12) |
| Liodakis[93] | 14 | 71.4 | nr | nr | 5.9(1.5) | nr | 12(86) | 0.4(11/13) |
| Liodakis[21] | 18 | 77.8 | nr | nr | nr | nr | 0 | 1.1(16/14) |
| Eralp[94] | 17 | 100.0 | 14/1/1/5 | 15/6/0/0 | 4.7(1,1-7.9) | 0.5(0.3-0.7) | 13(76) | 0.6(13/21) |
| Bas[95] | 20 | 100.0 | 10/4/1/0 | 10/5/0/0 | 5.5 (3-8) | 0.5(0.3-0.8) | 1(5) | 0.2(3/15) |
| Xu[88] | 12 | 100.0 | 8/3/1/0 | 9/3/0/0 | 4.1(3.0-6.0) | 0.6(0.5-0.7) | nr | 0.3(3/12) |
| **Summary** | **92** | **91.3** | **-** | **-** | **5.0(4.1-5.9)** | **0.6(0.5-0.9)** | **27(33)** | **0.7(60/87)** |
| **Tibia+ combined locking plate with Ilizarov** | | | | | | | | |
| Oh[96] | 10 | 100 | 10/0/0/0 | 6/3/1/0 | 2.6(nr) | 0.4(0.4-0.7) | 10(100)* | 1.1(11/10) |
| Gupta[97] | 14 | 100 | 14/0/0/0 | 8/6/0/0 | 4.5(0.6) | 0.7(0.1) | 14(100)* | 0.5(7/14) |
| Lu[98] | 12 | 100 | 12/0/0/0 | 0/8/4/0 | 3.7(nr) | 0.6(nr) | nr | 0.9(11/12) |
| **Sumamry** | **36** | **100** | **-** | **-** | **3.7(2.6-4.5)** | **0.6(0.4-0.7)** | **-** | **1.0(22/22)** |
| **Femur+ combined intramedullary nailing with Ilizarov** | | | | | | | | |
| Li[99] | 17 | 100.0 | 10/5/1/1 | 12/4/1/0 | nr | 0.6(0.5-0.8) | 0 | 1.6(27/17) |
| Wan[75] | 15 | 86.7 | 12/1/2/0 | 13/2/0/0 | 4.7(1.9) | 4.4(0.1) | 3(20) | 0.3(5/15) |
| Bas[95] | 19 | 100.0 | 13/1/2/3 | 16/3/0/0 | 5.3 (1.13-11.9) | 0.6(0.3-1) | 8(42) | 0.5(10/19) |
| Borzunov  [100] | 14 | 86.0 | nr | nr | 1.6(0.1) | 0.4(0.1) | 0 | 0.6(9/15) |
| **Summary** | **65** | **93.9** | **-** | **-** | **4.0(1.6-5.3)** | **1.5(0.4-4.4)** | **11(17)** | **0.8(51/66)** |

SD: standard deviation; EFT: external frame time, EFI: external frame index, nr: not reported

Bone result and functional result were evaluated by ASAMI (Association for the Study of the Method of Ilizarov).

*: active management of bone grafting was applied, and relevant studies were excluded when calculated the mean rate of bone grafting used.

**eReferences**

1. Paley D, Catagni M A, Argnani F, Villa A, Battista Benedetti G, Cattaneo R (1989) Ilizarov treatment of tibial nonunions with bone loss. *Clinical Orthopaedics and Related Research*, 241: 146-165. <https://doi.org/10.1097/00003086-198904000-00017>

2. Cattaneo R, Catagni M, Johnson E E (1992) The treatment of infected nonunions and segmental defects of the tibia by the methods of Ilizarov. *Clin Orthop Relat Res*(280): 143-152.

3. Cierny G, 3rd, Zorn K E (1994) Segmental tibial defects. Comparing conventional and Ilizarov methodologies. *Clin Orthop Relat Res*(301): 118-123.

4. Marsh J L, Prokuski L, Biermann J S (1994) Chronic infected tibial nonunions with bone loss. Conventional techniques versus bone transport. *Clin Orthop Relat Res*(301): 139-146.

5. Hosny G, Shawky M S (1998) The treatment of infected non-union of the tibia by compression-distraction techniques using the Ilizarov external fixator. *Int Orthop*, 22(5): 298-302. <https://doi.org/10.1007/s002640050264>

6. Ring D, Jupiter J B, Gan B S, Israeli R, Yaremchuk M J (1999) Infected nonunion of the tibia. *Clin Orthop Relat Res*(369): 302-311. <https://doi.org/10.1097/00003086-199912000-00032>

7. Paley D, Maar D C (2000) Ilizarov bone transport treatment for tibial defects. *J Orthop Trauma*, 14(2): 76-85. <https://doi.org/10.1097/00005131-200002000-00002>

8. Maini L, Chadha M, Vishwanath J, Kapoor S, Mehtani A, Dhaon B K (2000) The Ilizarov method in infected nonunion of fractures. *Injury*, 31(7): 509-517. <https://doi.org/10.1016/s0020-1383(00)00036-x>

9. Atesalp A S, Komurcu M, Basbozkurt M, Kurklu M (2002) The treatment of infected tibial nonunion with aggressive debridement and internal bone transport. *Mil Med*, 167(12): 978-981.

10. Bobroff G D, Gold S, Zinar D (2003) Ten year experience with use of Ilizarov bone transport for tibial defects. *Bull Hosp Jt Dis*, 61(3-4): 101-107.

11. Sen C, Kocaoglu M, Eralp L, Gulsen M, Cinar M (2004) Bifocal compression-distraction in the acute treatment of grade III open tibia fractures with bone and soft-tissue loss: a report of 24 cases. *J Orthop Trauma*, 18(3): 150-157. <https://doi.org/10.1097/00005131-200403000-00005>

12. Mekhail A O, Abraham E, Gruber B, Gonzalez M (2004) Bone transport in the management of posttraumatic bone defects in the lower extremity. *J Trauma*, 56(2): 368-378. <https://doi.org/10.1097/01.Ta.0000057234.48501.30>

13. Mahaluxmivala J, Nadarajah R, Allen P W, Hill R A (2005) Ilizarov external fixator: acute shortening and lengthening versus bone transport in the management of tibial non-unions. *Injury*, 36(5): 662-668. <https://doi.org/10.1016/j.injury.2004.10.027>

14. Robert Rozbruch S, Weitzman A M, Tracey Watson J, Freudigman P, Katz H V, Ilizarov S (2006) Simultaneous treatment of tibial bone and soft-tissue defects with the Ilizarov method. *J Orthop Trauma*, 20(3): 197-205. <https://doi.org/10.1097/00005131-200603000-00006>

15. Baruah R K (2007) Ilizarov methodology for infected non union of the Tibia: Classic circular transfixion wire assembly vs. hybrid assembly. *Indian J Orthop*, 41(3): 198-203. <https://doi.org/10.4103/0019-5413.33682>

16. Emara K M, Allam M F (2008) Ilizarov external fixation and then nailing in management of infected nonunions of the tibial shaft. *J Trauma*, 65(3): 685-691. <https://doi.org/10.1097/TA.0b013e3181569ecc>

17. Madhusudhan T R, Ramesh B, Manjunath K, Shah H M, Sundaresh D C, Krishnappa N (2008) Outcomes of Ilizarov ring fixation in recalcitrant infected tibial non-unions - a prospective study. *J Trauma Manag Outcomes*, 2(1): 6. <https://doi.org/10.1186/1752-2897-2-6>

18. Pirwani MA, Siddiqui MA, Soomro YH (2008) Management of infected non-union tibia by intercalary bone transport. *Pak J Surg*(24): 26-30.

19. Bumbasirević M, Tomić S, Lesić A, Milosević I, Atkinson H D (2010) War-related infected tibial nonunion with bone and soft-tissue loss treated with bone transport using the Ilizarov method. *Arch Orthop Trauma Surg*, 130(6): 739-749. <https://doi.org/10.1007/s00402-009-1014-6>

20. Wu C C (2011) Single-stage surgical treatment of infected nonunion of the distal tibia. *J Orthop Trauma*, 25(3): 156-161. <https://doi.org/10.1097/BOT.0b013e3181eaaa35>

21. Liodakis E, Kenawey M, Krettek C, Wiebking U, Hankemeier S (2011) Comparison of 39 post-traumatic tibia bone transports performed with and without the use of an intramedullary rod: the long-term outcomes. *Int Orthop*, 35(9): 1397-1402. <https://doi.org/10.1007/s00264-010-1094-5>

22. Chim H, Sontich J K, Kaufman B R (2011) Free tissue transfer with distraction osteogenesis is effective for limb salvage of the infected traumatized lower extremity. *Plast Reconstr Surg*, 127(6): 2364-2372. <https://doi.org/10.1097/PRS.0b013e318213a141>

23. Lin C C, Chen C M, Chiu F Y, Su Y P, Liu C L, Chen T H (2012) Staged protocol for the treatment of chronic tibial shaft osteomyelitis with Ilizarov's technique followed by the application of intramedullary locked nail. *Orthopedics*, 35(12): e1769-1774. <https://doi.org/10.3928/01477447-20121120-23>

24. Babar I U, Afsar S S, Gulzar M (2013) Treatment of segmental tibial bone loss by distraction osteogenesis. *Journal of Postgraduate Medical Institute*, 27(1): 78-82.

25. Sala F, Marinoni E, Miller A N, Pesenti G, Castelli F, Alati S, Coppadoro A, Capitani D (2013) Evaluation of an endoscopic procedure for the treatment of docking site nonunion. *J Orthop Trauma*, 27(10): 569-575. <https://doi.org/10.1097/BOT.0b013e31829484f6>

26. Xu K, Fu X, Li Y M, Wang C G, Li Z J (2014) A treatment for large defects of the tibia caused by infected nonunion: Ilizarov method with bone segment extension. *Ir J Med Sci*, 183(3): 423-428. <https://doi.org/10.1007/s11845-013-1032-9>

27. Feng Z H, Yuan Z, Jun L Z, Tao Z, Fa Z Y, Long M X (2013) Ilizarov method with bone segment extension for treating large defects of the tibia caused by infected nonunion. *Saudi Med J*, 34(3): 316-318.

28. Krappinger D, Irenberger A, Zegg M, Huber B (2013) Treatment of large posttraumatic tibial bone defects using the Ilizarov method: a subjective outcome assessment. *Arch Orthop Trauma Surg*, 133(6): 789-795. <https://doi.org/10.1007/s00402-013-1712-y>

29. Selim N M (2013) Ilizarov trifocal lengthening followed by intramedullary nailing for massive posttraumatic tibial bone defects. *Acta Orthop Belg*, 79(6): 706-710.

30. Atef A, El-Tantawy A (2014) Management of open infected comminuted tibial fractures using Ilizarov concept. *Eur J Orthop Surg Traumatol*, 24(3): 403-408. <https://doi.org/10.1007/s00590-013-1204-3>

31. Yin P, Zhang Q, Mao Z, Li T, Zhang L, Tang P (2014) The treatment of infected tibial nonunion by bone transport using the Ilizarov external fixator and a systematic review of infected tibial nonunion treated by Ilizarov methods. *Acta Orthop Belg*, 80(3): 426-435.

32. Morsy MA (2014) Unifocal bone transport in tibial nonunion using Ilizarov fixator. *Al-Azhar Asiut Med J* 2(12): 87-103.

33. Yin P, Zhang L, Li T, Zhang L, Wang G, Li J, Liu J, Zhou J, Zhang Q, Tang P (2015) Infected nonunion of tibia and femur treated by bone transport. *J Orthop Surg Res*, 10: 49. <https://doi.org/10.1186/s13018-015-0189-5>

34. Ajmera A, Verma A, Agrawal M, Jain S, Mukherjee A (2015) Outcome of limb reconstruction system in open tibial diaphyseal fractures. *Indian J Orthop*, 49(4): 429-435. <https://doi.org/10.4103/0019-5413.159638>

35. Azzam W, Atef A (2016) Our experience in the management of segmental bone defects caused by gunshots. *Int Orthop*, 40(2): 233-238. <https://doi.org/10.1007/s00264-015-2870-z>

36. Bernstein M, Fragomen A T, Sabharwal S, Barclay J, Rozbruch S R (2015) Does Integrated Fixation Provide Benefit in the Reconstruction of Posttraumatic Tibial Bone Defects? *Clin Orthop Relat Res*, 473(10): 3143-3153. <https://doi.org/10.1007/s11999-015-4326-6>

37. Fürmetz J, Soo C, Behrendt W, Thaller P H, Siekmann H, Böhme J, Josten C (2016) Bone Transport for Limb Reconstruction Following Severe Tibial Fractures. *Orthop Rev (Pavia)*, 8(1): 6384. <https://doi.org/10.4081/or.2016.6384>

38. Rohilla R, Wadhwani J, Devgan A, Singh R, Khanna M (2016) Prospective randomised comparison of ring versus rail fixator in infected gap nonunion of tibia treated with distraction osteogenesis. *Bone Joint J*, 98-b(10): 1399-1405. <https://doi.org/10.1302/0301-620x.98b10.37946>

39. Sadek A F, Laklok M A, Fouly E H, Elshafie M (2016) Two stage reconstruction versus bone transport in management of resistant infected tibial diaphyseal nonunion with a gap. *Arch Orthop Trauma Surg*, 136(9): 1233-1241. <https://doi.org/10.1007/s00402-016-2523-8>

40. Meleppuram J J, Ibrahim S (2017) Experience in fixation of infected non-union tibia by Ilizarov technique - a retrospective study of 42 cases. *Rev Bras Ortop*, 52(6): 670-675. <https://doi.org/10.1016/j.rboe.2016.11.008>

41. Tetsworth K, Paley D, Sen C, Jaffe M, Maar D C, Glatt V, Hohmann E, Herzenberg J E (2017) Bone transport versus acute shortening for the management of infected tibial non-unions with bone defects. *Injury*, 48(10): 2276-2284. <https://doi.org/10.1016/j.injury.2017.07.018>

42. Wang H, Wei X, Liu P, Fu Y H, Wang P F, Cong Y X, Zhang B F, Li Z, Lei J L, Zhang K *et al* (2017) Quality of life and complications at the different stages of bone transport for treatment infected nonunion of the tibia. *Medicine (Baltimore)*, 96(45): e8569. <https://doi.org/10.1097/md.0000000000008569>

43. Xu J, Zhong W R, Cheng L, Wang C Y, Wen G, Han P, Chai Y M (2017) The Combined Use of a Neurocutaneous Flap and the Ilizarov Technique for Reconstruction of Large Soft Tissue Defects and Bone Loss in the Tibia. *Ann Plast Surg*, 78(5): 543-548. <https://doi.org/10.1097/sap.0000000000000921>

44. Zhang Y, Wang Y, Di J, Peng A (2018) Double-level bone transport for large post-traumatic tibial bone defects: a single centre experience of sixteen cases. *Int Orthop*, 42(5): 1157-1164. <https://doi.org/10.1007/s00264-017-3684-y>

45. Wu Y, Yin Q, Rui Y, Sun Z, Gu S (2018) Ilizarov technique: Bone transport versus bone shortening-lengthening for tibial bone and soft-tissue defects. *J Orthop Sci*, 23(2): 341-345. <https://doi.org/10.1016/j.jos.2017.12.002>

46. Catagni M A, Azzam W, Guerreschi F, Lovisetti L, Poli P, Khan M S, Di Giacomo L M (2019) Trifocal versus bifocal bone transport in treatment of long segmental tibial bone defects. *Bone Joint J*, 101-b(2): 162-169. <https://doi.org/10.1302/0301-620x.101b2.Bjj-2018-0340.R2>

47. Rohilla R, Sharma P K, Wadhwani J, Beniwal R, Singh R, Devgan A, Rohilla S (2019) Prospective randomized comparison of quality of regenerate in distraction osteogenesis of ring versus monolateral fixator in patients with infected nonunion of the tibia using digital radiographs and CT. *Bone and Joint Journal*, 101-B(11): 1416-1422. <https://doi.org/10.1302/0301-620X.101B11.BJJ-2019-0189.R1>

48. Fahad S, Habib A A, Awais M B, Umer M, Rashid H U (2019) Infected Non-union of Tibia Treated with Ilizarov External Fixator: Our Experience. *Malays Orthop J*, 13(1): 36-41. <https://doi.org/10.5704/moj.1903.006>

49. Yikemu X, Tuxun A, Nuermaimaiti M, Abudukeyimu A, Shayiti A (2019) Effects of Vacuum Sealing Drainage Combined with Ilizarov Bone Transport Technique in the Treatment of Tibial Traumatic Osteomyelitis. *Med Sci Monit*, 25: 6864-6871. <https://doi.org/10.12659/MSM.915450>

50. Bakhsh K, Atiq Ur R, Zimri F K, Mohammad E, Ahmed W, Saaiq M (2019) Presentation and management outcome of tibial infected non-union with Ilizarov technique. *Pak J Med Sci*, 35(1): 136-140. <https://doi.org/10.12669/pjms.35.1.67>

51. Kinik H, Kalem M (2021) Ilizarov segmental bone transport of infected tibial nonunions requiring extensive debridement with an average distraction length of 9,5 centimetres. Is it safe? *Injury*, 52(8): 2425-2433. <https://doi.org/10.1016/j.injury.2019.12.025>

52. Jilani L Z, Shaan Z H, Ranjan R, Faizan M, Ahmad S, Asif N (2020) Management of complex non union of tibia using rail external fixator. *Journal of Clinical Orthopaedics and Trauma*, 11: S578-S584. <https://doi.org/10.1016/j.jcot.2019.12.016>

53. Li Y, Shen S, Xiao Q, Wang G, Yang H, Zhao H, Shu B, Zhuo N (2020) Efficacy comparison of double-level and single-level bone transport with Orthofix fixator for treatment of tibia fracture with massive bone defects. *Int Orthop*, 44(5): 957-963. <https://doi.org/10.1007/s00264-020-04503-2>

54. Li R, Zhu G, Chen C, Chen Y, Ren G (2020) Bone Transport for Treatment of Traumatic Composite Tibial Bone and Soft Tissue Defects: Any Specific Needs besides the Ilizarov Technique? *Biomed Res Int*, 2020: 2716547. <https://doi.org/10.1155/2020/2716547>

55. Wadhwani J, Rohilla R, Siwach R, Singh R, Devgan A, Vashishth S (2020) Comparison of Clinico-radiological Outcomes of Monolateral Fixator in Infected Non-union of Tibia Based on Bone Gap Quantification. *Indian J Orthop*, 54(4): 495-503. <https://doi.org/10.1007/s43465-020-00053-2>

56. Brauns A, Lammens J (2020) The challenge of the infected pilon tibial non-union: treatment with radical resection, bone transport and ankle arthrodesis. *Acta Orthop Belg*, 86(2): 335-341.

57. Abdou S A, Stranix J T, Daar D A, Mehta D D, McLaurin T, Tejwani N, Saadeh P B, Levine J P, Leucht P, Thanik V D (2020) Free Tissue Transfer with Distraction Osteogenesis and Masquelet Technique Is Effective for Limb Salvage in Patients with Gustilo Type IIIB Open Fractures. *Plast Reconstr Surg*, 145(4): 1071-1076. <https://doi.org/10.1097/prs.0000000000006696>

58. Abula A, Yushan M, Ren P, Abulaiti A, Ma C, Yusufu A (2020) Reconstruction of Soft Tissue Defects and Bone Loss in the Tibia by Flap Transfer and Bone Transport by Distraction Osteogenesis: A Case Series and Our Experience. *Ann Plast Surg*, 84(5S Suppl 3): S202-s207. <https://doi.org/10.1097/sap.0000000000002367>

59. Baruah R K, Baruah J P, Shyam-Sunder S (2020) Acute Shortening and Re-Lengthening (ASRL) in Infected Non-union of Tibia - Advantages Revisited. *Malays Orthop J*, 14(2): 47-56. <https://doi.org/10.5704/moj.2007.012>

60. Li R, Zeng C, Yuan S, Chen Y, Zhao S, Ren G H (2021) Free flap transplantation combined with Ilizarov bone transport for the treatment of severe composite tibial and soft tissue defects. *J Int Med Res*, 49(5): 3000605211017618. <https://doi.org/10.1177/03000605211017618>

61. Xu Y Q, Fan X Y, He X Q, Wen H J (2021) Reconstruction of massive tibial bone and soft tissue defects by trifocal bone transport combined with soft tissue distraction: experience from 31 cases. *BMC Musculoskelet Disord*, 22(1): 34. <https://doi.org/10.1186/s12891-020-03894-y>

62. Xiayimaierdan M, Huang J, Fan C, Cai F, Aihemaitijiang Y, Xie Z (2021) The efficiency of internal fixation with bone grafting at docking sites after bone transport for treatment of large segmental tibial bone defects. *Am J Transl Res*, 13(5): 5738-5745.

63. Huang Q, Ren C, Li M, Xu Y, Li Z, Lin H, Zhang K, Ma T (2021) Antibiotic calcium sulfate-loaded hybrid transport versus traditional Ilizarov bone transport in the treatment of large tibial defects after trauma. *J Orthop Surg Res*, 16(1): 568. <https://doi.org/10.1186/s13018-021-02723-9>

64. Huang Q, Xu Y, Lu Y, Ren C, Liu L, Li M, Wang Q, Li Z, Xue H, Zhang K *et al* (2022) Acute shortening and re-lengthening versus antibiotic calcium sulfate-loaded bone transport for the management of large segmental tibial defects after trauma. *J Orthop Surg Res*, 17(1): 219. <https://doi.org/10.1186/s13018-022-03109-1>

65. Rollo G, Luceri F, Falzarano G, Salomone C, Bonura E M, Popkov D, Ronga M, Pica G, Bisaccia M, Russi V *et al* (2021) Effectiveness of teriparatide combined with the Ilizarov technique in septic tibial non-union. *Med Glas (Zenica)*, 18(1): 287-292. <https://doi.org/10.17392/1280-21>

66. Kliushin N M, Burnashov S I, Mekki W A, Leonchuk D S, Sudnitsyn A S (2022) Treatment of postoperative tibial chronic osteomyelitis using bone transport techniques; an observational study. *J Clin Orthop Trauma*, 24: 101652. <https://doi.org/10.1016/j.jcot.2021.101652>

67. Hamiti Y, Yushan M, Yalikun A, Lu C, Yusufu A (2022) Matched comparative study of trifocal bone transport versus induced membrane followed by trifocal bone transport in the treatment of segmental tibial defects caused by posttraumatic osteomyelitis. *BMC Musculoskelet Disord*, 23(1): 572. <https://doi.org/10.1186/s12891-022-05501-8>

68. Rohilla R, Sharma P K, Wadhwani J, Das J, Singh R, Beniwal D (2022) Prospective randomized comparison of bone transport versus Masquelet technique in infected gap nonunion of tibia. *Arch Orthop Trauma Surg*, 142(8): 1923-1932. <https://doi.org/10.1007/s00402-021-03935-8>

69. Arfee S, Malik A T, Nehru A, Ali U, Arfee A, Arfee A A (2022) Clinicoradiological Comparison of Outcomes of LRS Fixator and Ilizarov in Infected Nonunion of Tibia Based on Bone Gap Quantification: An Original Research. *J Pharm Bioallied Sci*, 14(Suppl 1): S295-s297. <https://doi.org/10.4103/jpbs.jpbs_757_21>

70. Yushan M, Abulaiti A, Maimaiti X, Hamiti Y, Yusufu A (2022) Tetrafocal (three osteotomies) and pentafocal (four osteotomies) bone transport using Ilizarov technique in the treatment of distal tibial defect-preliminary outcomes of 12 cases and a description of the surgical technique. *Injury*, 53(8): 2880-2887. <https://doi.org/10.1016/j.injury.2022.06.006>

71. Abulaiti A, Liu Y, Cai F, Liu K, Abula A, Maimaiti X, Ren P, Yusufu A (2022) Bone Defects in Tibia Managed by the Bifocal vs. Trifocal Bone Transport Technique: A Retrospective Comparative Study. *Front Surg*, 9: 858240. <https://doi.org/10.3389/fsurg.2022.858240>

72. Krishnan A, Pamecha C, Patwa J J (2006) Modified Ilizarov technique for infected nonunion of the femur: the principle of distraction-compression osteogenesis. *J Orthop Surg (Hong Kong)*, 14(3): 265-272. <https://doi.org/10.1177/230949900601400307>

73. Saridis A, Panagiotopoulos E, Tyllianakis M, Matzaroglou C, Vandoros N, Lambiris E (2006) The use of the Ilizarov method as a salvage procedure in infected nonunion of the distal femur with bone loss. *J Bone Joint Surg Br*, 88(2): 232-237. <https://doi.org/10.1302/0301-620x.88b2.16976>

74. Arora S, Batra S, Gupta V, Goyal A (2012) Distraction osteogenesis using a monolateral external fixator for infected non-union of the femur with bone loss. *J Orthop Surg (Hong Kong)*, 20(2): 185-190. <https://doi.org/10.1177/230949901202000210>

75. Wan J, Ling L, Zhang X S, Li Z H (2013) Femoral bone transport by a monolateral external fixator with or without the use of intramedullary nail: a single-department retrospective study. *Eur J Orthop Surg Traumatol*, 23(4): 457-464. <https://doi.org/10.1007/s00590-012-1008-x>

76. Agrawal H K, Garg M, Singh B, Jaiman A, Khatkar V, Khare S, Batra S, Sharma V K (2016) Management of complex femoral nonunion with monorail external fixator: A prospective study. *J Clin Orthop Trauma*, 7(Suppl 2): 191-200. <https://doi.org/10.1016/j.jcot.2016.02.013>

77. Liu C, Zhang X, Zhang X, Li Z, Xu Y, Liu T (2019) Bone transport with a unilateral external fixator for femoral infected nonunion after intramedullary nailing fixation: A case control study. *Medicine (Baltimore)*, 98(20): e15612. <https://doi.org/10.1097/md.0000000000015612>

78. Sen C, Demirel M, Sağlam Y, Balcı H I, Eralp L, Kocaoğlu M (2019) Acute shortening versus bone transport for the treatment of infected femur non-unions with bone defects. *Injury*, 50(11): 2075-2083. <https://doi.org/10.1016/j.injury.2019.08.021>

79. Bakhsh K, Zimri F K, Atiq Ur R, Mohammad E, Saaiq M (2019) Outcome of complex non-unions of femoral fractures managed with Ilizarov method of distraction osteogenesis. *Pak J Med Sci*, 35(4): 1055-1059. <https://doi.org/10.12669/pjms.35.4.244>

80. Rohilla R, Sharma P K, Dua M, Singh R, Beniwal D, Khokhar A (2022) Outcome of monolateral rail fixator in infected nonunion of femur diaphysis developing after intramedullary fixation. *Eur J Orthop Surg Traumatol*. <https://doi.org/10.1007/s00590-022-03275-2>

81. Hutson J J, Jr., Dayicioglu D, Oeltjen J C, Panthaki Z J, Armstrong M B (2010) The treatment of gustilo grade IIIB tibia fractures with application of antibiotic spacer, flap, and sequential distraction osteogenesis. *Ann Plast Surg*, 64(5): 541-552. <https://doi.org/10.1097/SAP.0b013e3181cf9fb5>

82. Spiegl U, Pätzold R, Friederichs J, Hungerer S, Militz M, Bühren V (2013) Clinical course, complication rate and outcome of segmental resection and distraction osteogenesis after chronic tibial osteitis. *Injury*, 44(8): 1049-1056. <https://doi.org/10.1016/j.injury.2013.05.003>

83. Peng J, Min L, Xiang Z, Huang F, Tu C, Zhang H (2015) Ilizarov bone transport combined with antibiotic cement spacer for infected tibial nonunion. *Int J Clin Exp Med*, 8(6): 10058-10065.

84. van Niekerk A H, Birkholtz F F, de Lange P, Tetsworth K, Hohmann E (2017) Circular external fixation and cemented PMMA spacers for the treatment of complex tibial fractures and infected nonunions with segmental bone loss. *J Orthop Surg (Hong Kong)*, 25(2): 2309499017716242. <https://doi.org/10.1177/2309499017716242>

85. Hamiti Y, Yushan M, Lu C, Yusufu A (2021) Reconstruction of massive tibial defect caused by osteomyelitis using induced membrane followed by trifocal bone transport technique: a retrospective study and our experience. *BMC Surg*, 21(1): 419. <https://doi.org/10.1186/s12893-021-01421-x>

86. Liu K, Liu Y, Cai F, Fan C, Ren P, Yusufu A (2022) Efficacy comparison of trifocal bone transport using unilateral external fixator for femoral and tibial bone defects caused by infection. *BMC Surg*, 22(1): 141. <https://doi.org/10.1186/s12893-022-01586-z>

87. Khaled A, El-Gebaly O, El-Rosasy M (2022) Masquelet-Ilizarov technique for the management of bone loss post debridement of infected tibial nonunion. *Int Orthop*, 46(9): 1937-1944. <https://doi.org/10.1007/s00264-022-05494-y>

88. Xu Y, Ma T, Ren C, Li M, Lu Y, Sun L, Huang Q, Wang Q, Xue H, Li Z *et al* (2022) Treatment of tibial large bone defects: A comparative study of bone transport over an intramedullary nail in combination with antibiotic-impregnated calcium sulphate versus bone transport alone with antibiotic-impregnated calcium sulphate. *Injury*. <https://doi.org/10.1016/j.injury.2022.09.042>

89. Song H R, Kale A, Park H B, Koo K H, Chae D J, Oh C W, Chung D W (2003) Comparison of internal bone transport and vascularized fibular grafting for femoral bone defects. *J Orthop Trauma*, 17(3): 203-211. <https://doi.org/10.1097/00005131-200303000-00009>

90. Sen C, Akgül T, Tetsworth K D, Balci H İ, Yildiz F, Necmettin T (2020) Combined Technique for the Treatment of Infected Nonunions of the Distal Femur with Bone Loss: Short Supracondylar Nail-Augmented Acute Shortening/Lengthening. *Journal of Orthopaedic Trauma*, 34(9): 476-481. <https://doi.org/10.1097/BOT.0000000000001764>

91. Peng C, Liu K, Tian Q, Tusunniyazi M, Kong W, Luan H, Liu X, Zhao Y (2022) Evaluation of complications associated with bifocal bone transport as treatment for either proximal, intermediate or distal femoral defects caused by infection: outcome analysis of 76 patients. *BMC Musculoskelet Disord*, 23(1): 132. <https://doi.org/10.1186/s12891-022-05078-2>

92. Oh C W, Song H R, Roh J Y, Oh J K, Min W K, Kyung H S, Kim J W, Kim P T, Ihn J C (2008) Bone transport over an intramedullary nail for reconstruction of long bone defects in tibia. *Arch Orthop Trauma Surg*, 128(8): 801-808. <https://doi.org/10.1007/s00402-007-0491-8>

93. Liodakis E, Kenawey M, Krettek C, Ettinger M, Jagodzinski M, Hankemeier S (2011) Segmental transports for posttraumatic lower extremity bone defects: are femoral bone transports safer than tibial? *Arch Orthop Trauma Surg*, 131(2): 229-234. <https://doi.org/10.1007/s00402-010-1129-9>

94. Eralp L, Kocaoǧlu M, Polat G, Baş A, Dirican A, Azam M E (2012) A comparison of external fixation alone or combined with intramedullary nailing in the treatment of segmental tibial defects. *Acta Orthopaedica Belgica*, 78(5): 652-659.

95. Bas A, Daldal F, Eralp L, Kocaoglu M, Uludag S, Sari S (2020) Treatment of Tibial and Femoral Bone Defects With Bone Transport Over an Intramedullary Nail. *J Orthop Trauma*, 34(10): e353-e359. <https://doi.org/10.1097/bot.0000000000001780>

96. Oh C W, Apivatthakakul T, Oh J K, Kim J W, Lee H J, Kyung H S, Baek S G, Jung G H (2013) Bone transport with an external fixator and a locking plate for segmental tibial defects. *Bone Joint J*, 95-b(12): 1667-1672. <https://doi.org/10.1302/0301-620x.95b12.31507>

97. Gupta S, Malhotra A, Mittal N, Garg S K, Jindal R, Kansay R (2018) The management of infected nonunion of tibia with a segmental defect using simultaneous fixation with a monorail fixator and a locked plate. *Bone Joint J*, 100-b(8): 1094-1099. <https://doi.org/10.1302/0301-620x.100b8.Bjj-2017-1442.R1>

98. Lu Y, Ma T, Ren C, Li Z, Sun L, Xue H, Li M, Zhang K, Zhang C, Wang Q (2020) Treatment of segmental tibial defects by bone transport with circular external fixation and a locking plate. *J Int Med Res*, 48(4): 300060520920407. <https://doi.org/10.1177/0300060520920407>

99. Li Z, Zhang X, Duan L, Chen X (2009) Distraction osteogenesis technique using an intramedullary nail and a monolateral external fixator in the reconstruction of massive postosteomyelitis skeletal defects of the femur. *Can J Surg*, 52(2): 103-111.

100. Borzunov D Y, Kolchin S N (2022) Nonunion of the femoral shaft associated with limb shortening treated with a combined technique of external fixation over an intramedullary nail versus the Ilizarov method. *Arch Orthop Trauma Surg*, 142(9): 2185-2192. <https://doi.org/10.1007/s00402-021-03804-4>
