# Supplementary material for: Ilizarov method and its combined methods in the treatment of long bone defects of the lower extremity: systematic review and meta-analysis
Source: BMC Musculoskelet Disord. 2023 Nov 16;24:891. doi: 10.1186/s12891-023-07001-9 (PMC10652567; doi:10.1186/s12891-023-07001-9)
Supplement: Supplementary file 2 — Additional file 2. Pooled estimation and comparative analysis of interested outcomes of Ilizarov method by locations. [file 12891_2023_7001_MOESM2_ESM.docx]

**Additional file 2:** Pooled estimation and comparative analysis of interested outcomes of Ilizarov method by locations.

|  | **Tibia** | | | | **Femur** | | | Comparison |
| --- | --- | --- | --- | --- | --- | --- | --- | --- |
|  | Studies (n)  (Patients, n) | Pooled effect  size (%, 95% CI) | Heterogeneity  (*I*^2^, %) |  | Studies (n)  (Patients, n) | Pooled effect size (%, 95% CI) | Heterogeneity  (*I*^2^, %) |  |
| Union rate | 83 (2069/2159) | 99.29  (98.67, 99.86) | 2.3 |  | 11  (242/250) | 98.81  (96.78, 100.00) | 0.0 | RR = 0.99  (0.97, 1.01) |
| Excellent rate in bone result | 54  (842/1472) | 60.58  (55.78, 65.80) | 76.3 |  | 13  (143/250) | 62.48  (52.70, 74.07) | 73.7 | RR = 1.00  (0.89, 1.12) |
| Pin infection | 54  (693/1363) | 49.44  (42.01, 58.19) | 89.5 |  | 11  (154/250) | 62.38  (51.70, 75.27) | 69.8 | RR =0.83  (0.74, 0.92) |
| Pin loose | 14  (37/406) | 6.80  (4.46, 9.13) | 0.0 |  | 4  (21/116) | 17.00  (9.27, 24.72) | 0.0 | RR =0.50  (0.31, 0.83) |
| Pain | 12  (55/342) | 13.48  (3.72, 23.24) | 85.9 |  | 4  (15/78) | 15.41  (1.79, 29.03) | 72.5 | RR = 0.83  (0.50, 1.40) |
| Refracture* | 37  (46/1089) | 2.25  (1.16, 3.33) | 0.0 |  | 6  (6/124) | 2.58  (0.00, 6.07) | 0.0 | RR = 0.87  (0.38, 2.00) |
| Limb discrepancy  (> 2 cm) | 20  (46/590) | 4.58  (2.55, 6.61) | 45.1 |  | 3  (6/76) | 5.34  (0.36, 10.33) | 16.5 | RR = 0.99  (0.44, 2.23) |
| Malalignment  (> 5°) | 33  (93/768) | 16.68  (12.87, 21.61) | 54.3 |  | 6  (34/121) | 31.72  (22.14, 45.45) | 43.1 | RR = 0.43  (0.31, 0.61) |
| Joint stiffness | 39  (222/974) | 23.04  (15.82, 30.52) | 96.0 |  | 8  (66/186) | 30.87  (14.11, 47.63) | 88.9 | RR = 0.64  (0.51, 0.80) |
| Recurrent infection | 43  (73/1098) | 1.91  (0.85, 2.97) | 52.4 |  | 4  (4/111) | 3.24  (0.00, 7.19) | 0.0 | RR = 1.84  (0.69, 4.95) |
| Amputation | 63  (19/1583) | 0.28  (0.00, 0.91) | 0.0 |  | 2  (1/161) | 0.20  (0.00, 2.41) | 0.0 | - |

RR: relative risk

* either the regenerate bone or at the docking site
